# The Polyphenolic Profile and Antioxidant Activity of Five Vegetal Extracts with Hepatoprotective Potential

**DOI:** 10.3390/plants11131680

**Published:** 2022-06-24

**Authors:** Liliana Costea, Carmen Lidia Chițescu, Rica Boscencu, Manuela Ghica, Dumitru Lupuliasa, Dragoș Paul Mihai, Teodora Deculescu-Ioniță, Ligia Elena Duțu, Maria Lidia Popescu, Emanuela-Alice Luță, George Mihai Nițulescu, Octavian Tudorel Olaru, Cerasela Elena Gîrd

**Affiliations:** 1 Faculty of Pharmacy, “Carol Davila” University of Medicine and Pharmacy, Traian Vuia 6, 020956 Bucharest, Romania; rica.boscencu@umfcd.ro (R.B.); manuela.ghica@umfcd.ro (M.G.); dumitru.lupuliasa@umfcd.ro (D.L.); teodora.deculescu-ionita@umfcd.ro (T.D.-I.); ligia.dutu@umfcd.ro (L.E.D.); maria.popescu@umfcd.ro (M.L.P.); emanuela.luta@drd.umfcd.ro (E.-A.L.); george.nitulescu@umfcd.ro (G.M.N.); octavian.olaru@umfcd.ro (O.T.O.); cerasela.gird@umfcd.ro (C.E.G.); 2 Faculty of Medicine and Pharmacy, “Dunărea de Jos”, University of Galați, 35 A.I. Cuza Str., 800010 Galați, Romania

**Keywords:** antioxidant activity, hepatoprotective effect, polyphenolic profile, phytochemicals, vegetal extracts, UHPLC–HRMS/MS, molecular docking

## Abstract

Oxidative stress is among the major triggers for many important human functional disorders, which often lead to various metabolic or tissue diseases. The aim of the study is to obtain five standardized vegetal extracts (*Cynarae extractum*—CE, *Rosmarini extractum*—RE, *Taraxaci extractum*—TE, *Cichorii extractum*—CHE, and *Agrimoniae extractum*—AE) that contain active principles with an essential role in protecting liver cells against free radicals and quantify their antioxidant actions. The compounds of therapeutic interest from the analyzed extracts were identified and quantified using the UHPLC–HRMS/MS technique. Thus, the resulting identified compounds were 28 compounds in CE, 48 compounds in RE, 39 compounds in TE, 43 compounds in CHE, and 31 compounds in AE. These compounds belong to the class of flavonoids, isoflavones, phenolic acids and dicarboxylic acids, depsides, diterpenes, triterpenes, sesquiterpenes, proanthocyanidins, or coumarin derivatives. From the major polyphenolic compounds quantified in all the extracts analyzed by UHPLC–HRMS/MS, considerable amounts have been found for chlorogenic acid (619.8 µg/g extract for TE–2032.4 µg/g extract for AE), rutoside (105.1 µg/g extract for RE–1724.7 µg/g extract for AE), kaempferol (243 µg/g extract for CHE–2028.4 µg/g extract for CE), and for naringenin (383 µg/g extract for CHE–1375.8 µg/g extract for AE). The quantitative chemical analysis showed the highest content of total phenolic acids for AE (24.1528 ± 1.1936 g chlorogenic acid/100 g dry extract), the highest concentration of flavones for RE (6.0847 ± 0.3025 g rutoside/100 g dry extract), and the richest extract in total polyphenols with 31.7017 ± 1.2211 g tannic acid equivalent/100 g dry extract for AE. Several methods (DPPH, ABTS, and FRAP) have been used to determine the in vitro total antioxidant activity of the extracts to evaluate their free radical scavenging ability, influenced by the identified compounds. As a result, the correlation between the content of the polyphenolic compounds and the antioxidant effect of the extracts has been demonstrated. Statistically significant differences were found when comparing the antiradical capacity within the study groups. Although all the analyzed extracts showed good IC50 values, which may explain their antihepatotoxic effects, the highest antioxidant activity was obtained for *Agrimoniae extractum* (IC50_ABTS_ = 0.0147 mg/mL) and the lowest antioxidant activity was obtained for *Cynarae extractum* (IC50_ABTS_ = 0.1588 mg/mL). Furthermore, the hepatoprotective potential was evaluated in silico by predicting the interactions between the determined phytochemicals and key molecular targets relevant to liver disease pathophysiology. Finally, the evaluation of the pharmacognostic and phytochemical properties of the studied extracts validates their use as adjuvants in phytotherapy, as they reduce oxidative stress and toxin accumulation and thus exert a hepatoprotective effect at the cellular level.

## 1. Introduction

Oxidative stress is characterized by a disruption in the balance of antioxidants and prooxidants at the cellular level as well as a failure of the endogenous antioxidants to provide protection at this level, resulting in pathophysiologic alteration or disorders inducing various types of diseases (diabetes, liver disease, rheumatoid arthritis, cardiovascular disease, neurovegetative disease, cancer, etc.) [1].

Non-alcoholic fatty liver disease (NAFLD) is a major cause of liver disease and is defined as an excessive accumulation of fat, especially triglycerides, in liver cells. It is closely linked to the metabolic syndrome and its associated conditions (diabetes and dyslipidemia) [2,3,4]. So far, there has been no ideal pharmacological treatment for NAFLD [5], with the current recommendations comprising a generally healthier lifestyle, including a healthy diet, intense physical activity, and weight loss [5,6,7,8]. Due to the poor adherence to this type of treatment, especially in the case of long-term weight loss diets, with the potential to induce side effects on the liver cells [5,7,9], there is a growing interest regarding the identification of new therapeutic agents for the treatment and/or prevention of NAFLD progression. Since conventional pharmacological therapies lead to side effects, there is a need to find vegetal products and combinations of chemically characterized plant extracts with good safety profiles containing polyphenolic compounds, which are known for their antioxidant actions.

The plant kingdom offers a variety of vegetal sources that contain secondary metabolites with hepatoprotective action, making them potential candidates for a new phytotherapeutic formulation. Among the plant sources associated in liver diseases for their hepatoprotective activity, the following can be listed: *Cynarae folium* (artichoke leaves), *Rosmarini folium* (rosemary leaves), *Taraxaci herba* (dandelion aerial parts), *Cichorii herba* (common chicory aerial parts), and *Agrimoniae herba* (agrimony aerial parts).

*Cynara scolymus* L. is known for its therapeutic qualities due to its choleretic-cholagogue, hepatoprotective, antioxidant, and cholesterol-lowering effects, through the phytocomplex represented by caffeic acid, chlorogenic acid (isomers), cynarin, cynaropicrin, and cynaroside. The herbal product *Cynarae folium* is frequently associated in phytotherapy [10,11,12,13,14,15,16].

The leaves of *Rosmarinus officinalis* L. are used in phytotherapy, and the volatile oil is used in aromatherapy. *Rosmarini folium* stimulates cerebral circulation and microcirculation (due to rosmarinic acid) and has cholesterol-lowering and choleretic effects (due to phenolcarboxylic acids), diuretic action (due to flavones and triterpenes), antispasmodic action (by flavones), and antioxidant effects (due to rosmarinic acid, caffeic acid, chlorogenic acid, and flavonoid derivatives). *Rosmarini aetheroleum* is used in aromatherapy for its tonic, expectorant, antifungal, antibacterial, and mucolytic effects [17,18,19,20,21,22,23].

*Taraxacum officinale* L. through the two types of vegetal products *Taraxaci radix et herba* is used as a bitter tonic agent, stimulant of pancreatic secretion, laxative (due to bitter principles and mucilage), diuretic (due to flavones), cholagogue (due to bitter tonics), a cholesterol-lowering, and a lipid-lowering agent (due to bitters, flavones, and phenolic acids), as well as a detoxifying and hepatoprotective agent (due to the phytocomplex) [24,25,26,27,28,29,30].

The root and aerial parts from the species *Cichorium intybus* L. are used in phytotherapy. It has a tonic action on digestion (due to bitter principles), diuretic and depurative effects (due to flavones, triterpenes, and bitter principles), cholagogue effects (due to bitters and total phenolic acids), cholesterol-lowering effects (due to bitter principles), and hepatoprotective effects (due to phytocomplex) [31,32,33,34,35,36,37].

The aerial parts harvested from the species *Agrimonia eupatoria* L. are used in phytotherapy for the treatment of gastrointestinal diseases (gastritis, enteritis, peptic ulcer disease, and nonspecific diarrhea). Additionally, they are used in the treatment of biliary or hepatic disorders [38,39,40,41,42,43,44].

Based on the data from the scientific literature, this paper presents the research that was carried out to obtain vegetal extracts that can be used in phytotherapeutic formulations with hepatoprotective properties. The obtained extracts were characterized by qualitative and quantitative chemical analysis (quantitative analysis of phytochemicals such as flavones spectrophotometric assays, phenolcarboxylic acids colorimetric assays, total polyphenols spectrophotometric assays, or identification and quantification of the secondary metabolites by HPLC). Additionally, the antioxidant effect was determined by the DPPH, ABTS, and FRAP methods.

The hepatoprotective potential was evaluated in silico by predicting the interactions between the determined phytochemicals and key molecular targets relevant to liver disease pathophysiology. Thus, various polyphenols were assessed through molecular docking as potential inhibitors of the cytochrome P450 2E1 (CYP2E1) isoform, tumor necrosis factor alpha (TNF-α), and as allosteric activators of glutathione peroxidase 4 (GPx4). CYP2E1 is responsible for converting polyunsaturated fatty acids and exogenous compounds to toxic metabolites, and its inhibition can alleviate hepatotoxicity [45]. TNF-α is a cytokine that causes inflammation, oxidative stress, and hepatocyte apoptosis [46], whereas GPx4 is an antioxidant enzyme that prevents hepatocellular degeneration by suppressing lipid peroxidation and inflammation [47].

## 2. Results

### 2.1. Preparation of Vegetal Extracts

The obtained extracts were in the form of dry powders, with a uniform dispersion by homogenization, and they sum up the organoleptic particularities (color, smell, and taste) specific to the plant source from which they were obtained. The extraction yield varied depending on the vegetable raw material used and the concentration of alcohol used for the extraction. Thus, the yield was 12.54% for CE (*Cynarae extractum*), 12.84% for RE (*Rosmarini extractum*), 24.31% for TE (*Taraxaci extractum*), 12.51% for CHE (*Cichorii extractum*), and 13.23% for AE (*Agrimoniae extractum*).

### 2.2. Quantitative and Qualitative Chemical Analysis

The results obtained from the quantitative chemical determinations are presented in Table 1.

The quantitative chemical analysis showed that the extracts have a variable amount of secondary metabolites. AE is the richest in TPA, total phenolic acid content (expressed in chlorogenic acid), with a concentration of 24.1528 ± 1.1936 g phenolic acids/100 g dry extract, while CE has a concentration of only 1.7389 ± 0.0904 g chlorogenic acid/100 g dry extract. It was also observed that RE, TE, and CHE have appreciable amounts in these derivatives. Regarding the total flavonoid content, RE has the highest concentration of 6.0847 ± 0.3025 g flavones/100 g dry extract expressed in rutoside equivalents and TE has the lowest concentration of only 1.9019 ± 0.1080 g rutoside/100 g dry extract. The total phenolic content varies between 5.7627 ± 0.6946 g tannic acid equivalent for CE and 31.7017 ± 1.2211 g tannic acid equivalent for AE, which is the richest in total polyphenols, with a strong antioxidant role (Table 1).

### 2.3. UHPLC–HRMS/MS Analysis

The spectrum of the identified and quantified compounds is quantitatively dependent on the type of the extract used, the identified compounds being in the class of flavonoids, phenolcarboxylic acids, and sesquiterpene derivatives. The analysis showed that 28 compounds were identified in CE (Table 2, Figure 1 and Figure 2, Supplementary Materials Figure S1), 48 compounds in RE (Table 3, Figure 3, Supplementary Materials Figures S2–S5), 39 compounds in TE (Table 4, Figure 4, Supplementary Materials Figures S6–S8), 43 compounds in CHE (Table 5, Figure 5 and Figure 6, Supplementary Materials Figures S9–S11), and 31 compounds in AE (Table 6, Figure 7, Supplementary Materials Figures S12–S14).

According to our results, rutoside was identified in all extracts except AE; apigenin and kaempferol in all types of extracts; chlorogenic acid and caffeic acid in all extracts except CHE (where only chlorogenic acid was identified with one of its isomers); vitexin was identified in CE, TE, CHE, and AE; cynarine, scolimoside, and cynaropicrin were identified in CE; cichorin in CE, RE, and CHE; cynaroside and cynarotrioside in TE, CHE, and AE; catechin and epicatechin in CHE and AE; azelaic acid in all extracts; biochanin A in RE, CHE, and AE; genistin and daidzin in CHE; genistin in TE and AE; and condensed procyanidins (proanthocyanidins) in TE, CHE, and AE.

**Figure 2:**
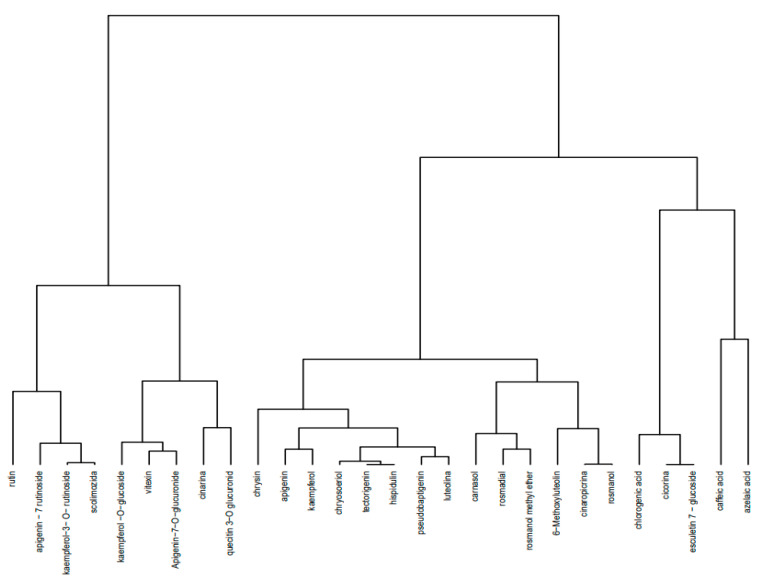
Dendrogram for **CE** compounds representative of the profile of polyphenolic derivatives obtained by the hierarchical grouping method Ward-HCA (Hierarchical Cluster Analysis).

The artichoke extract composition’s dendrogram (Figure 2) shows the disposition of the identified compounds in four clusters. The first cluster contains a group of 9 compounds of the flavonoid class, the second one contains a group of 14 compounds represented by flavonoid aglycones (in this group there are 2 diterpene derivatives—carnosol and rosmanol, and 1 sesquiterpene compound—cynaropicrin), the third one contains a group of phenolic compounds such as chlorogenic acid and 2 coumarin derivatives, and the last one contains 2 phenolic acids—caffeic and azelaic acid.

The dendrogram of the rosemary extract’s chemical composition (Supplementary Materials Figure S5) reveals the disposition of the identified compounds in five clusters. The first cluster contains a group of 12 compounds of the flavonoid class (the heteroside forms); the second one contains a group of 29 compounds represented by flavonoid aglycones and glycosides, phenolic acids, and derivatives with an isoflavan nucleus; the third one contains a group of 5 compounds; and the fourth and fifth ones contain 1 compound each.

The dendrogram of the dandelion extract’s chemical composition (Supplementary Materials Figure S8) shows the disposition of the identified compounds in five clusters. The first cluster contains a group of 15 compounds from the flavonoid class, the second one contains 16 compounds represented by flavonoid aglycones or glycosides and phenolic acids, the third one contains a group of 3 compounds, and the fourth and fifth ones contain compounds from the category of phenolic acids.

**Figure 6:**
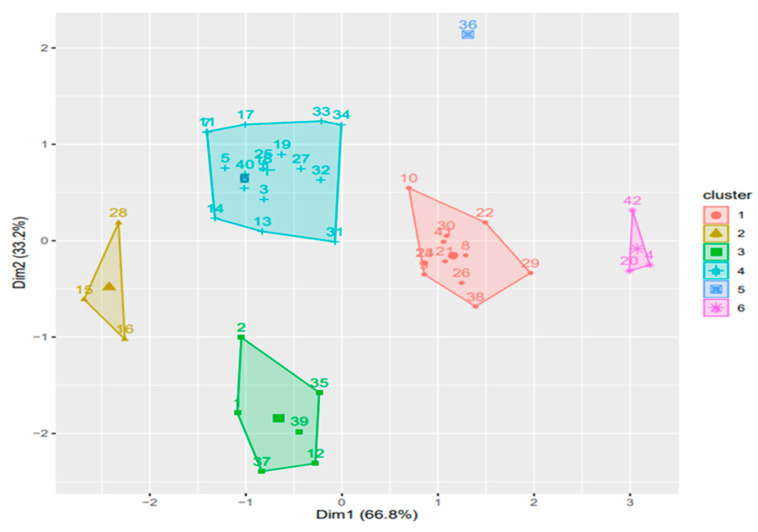
Diagram for **CHE** compounds representative of the profile of polyphenolic derivatives obtained by the hierarchical grouping methods Ward and HCA (Hierarchical Cluster Analysis; another illustration of the dendrogram for CHE-flavonoids, phenolic acids, dicarboxylic acids, and other representative compounds).

The dendrogram of the chicory extract’s chemical composition (Figure 6) shows the disposition of the identified compounds in six clusters. The first one contains a single compound, the oleanolic acid, a triterpene compound, while the other clusters contain groups of 3 (procyanidin, rutin, and kaempferol-3-O-rutinoside), 11 (isorhamnetin-3-O-glucoside, cynarine, chrysoeriol-7-glucoside, chicoric acid, genistin, vitexin, apigetrin, cynaroside, apigenin-7-O-glucuronide, hyperoside, and kaempferol), 18 (abscisic acid, quercetin, ellagic acid, biochanin A, chrysin, 2′,6-dihydroxyflavone, irisolidone, pratensein, chrysoeriol, apigenin, kaempferol-O-glucoside, luteolin, rosmadial, rosmanol methyl ether, tricin, rosmanol, daidzin, and rosmarinic acid), 3 (azelaic acid, *p*-coumaric acid, and syringic acid), and 6 (caftaric acid, chlorogenic acid, cichorin, neochlorogenic acid, catechin, and epicatechin) polyphenolic compounds, respectively.

The agrimony extract dendrogram (Supplementary Materials Figure S14) shows the disposition of the identified compounds in five clusters, the most representative being the third one, in which 16 compounds of the polyphenol class are grouped.

From a quantitative point of view, 13 compounds were quantified in CE, 17 in RE, 18 in TE, 19 in CHE, and 19 in AE (Table 7).

A horizontal analysis of the results shows that AE is rich in catechin (11,854.8 µg/g extract), while appreciable amounts (but half the concentration for AE) were also quantified in CHE (5885.7 µg/g extract) and TE (5883.4 µg/g extract). Additionally, we observed that caffeic acid was not quantifiable in CHE; genistin, an isoflavone, was quantified in appreciable amounts in AE (5514.9 µg/g extract); chlorogenic acid was quantified in all the types of extract, with concentrations ranging from 619.8 µg/g TE extract to 2032.4 µg/g extract in AE; the hyperoside was quantified in an appreciable amount in AE (15,431.3 µg/g extract), but was not quantified in CE and RE; apigenin, rutoside, ellagic acid, and naringenin were quantified in different amounts in all the types of extract; and quercetol was predominant in AE (4958.05 µg/g extract).

### 2.4. Antioxidant Activity

The antioxidant effects induced by the tested extracts were directly correlated with the concentration of secondary metabolites (Table 1). Comparing the obtained results, it was found that the most intense antioxidant actions were induced by AE (lowest IC50 value by all three methods, compared to the other extracts), which is justified by the higher content of polyphenols in this type of extract (Table 8). It can also be noted that, of all the extracts analyzed, the IC50/EC50 values obtained for AE are much closer to the antioxidant values of the standard used (ascorbic acid), which accentuates the superior antioxidant action of AE compared to the other samples. All the analyzed extracts contain significant amounts of total polyphenols, with high concentrations for CHE, RE, and AE, and moderate concentrations for CE and TE. In addition, high values of phenolcarboxylic acids were identified in their composition: high concentrations for RE and AE and moderate concentrations for the other extracts (Table 8).

Therefore, it is particularly important to evaluate the correlation between the total polyphenol content and the antioxidant effect of the extracts and the correlation between TPA and antioxidant action.

Given that the antioxidant effect was determined by three methods (DPPH, ABTS, and FRAP), it was analyzed using One-way ANOVA if there were statistically significant differences between the results of the antioxidant action obtained by the three methods (Supplementary Materials Table S1). It was observed that there are no statistically significant differences at the 95% significance threshold between the three methods applied to evaluate the antioxidant effect (*p* = 0.1170, *p* > 0.05-Table 9).

The data in Supplementary Materials Table S2 show the correlation between the methodologies used in this study (DPPH, ABTS, and FRAP) for the evaluation of the antioxidant action in the analyzed extracts. The Pearson coefficient (r) calculated for each set of data pairs obtained by the different methods shows values above 0.900, which presents a very strong correlation between the experimentally applied methodologies (ABTS vs. DPPH: r = 0.995, *p* = 0.000; ABTS vs. FRAP: r = 0.964, *p* = 0.008; DPPH vs. FRAP: r = 0.982, *p* = 0.003).

The boxplot diagram showing the distribution and spread of IC50 values between different methods is presented in Supplementary Materials Figure S15.

Besides revealing a very well-correlated antioxidant activity of the extracts, the Pearson coefficient (r > 0.900) as well as the coefficients of determination (R^2^ > 0.900) show that the results are not significantly influenced by methodological errors or other interferences related to the principles used in the determinations (Table 10).

Subsequently, the statistically significant differences within the same method were analyzed by dispersion analysis. For the DPPH method, the results obtained by statistical validation using One-Way ANOVA showed statistically significant differences (*p* < 0.05; Table 11) between the free radical reduction effects of the plant extracts. After comparing the averages between groups for the DPPH method with the post hoc tests, statistically significant differences were observed, expressed by the *p* value as follows: *Cynara* vs. *Rosmarinus* (*p* = 0.004), *Cynara* vs. *Agrimonia* (*p* = 0.000), *Cynara* vs. *Taraxacum* (*p* = 0.045), *Cynara* vs. *Cichorium* (*p* = 0.002), *Rosmarinus* vs. *Taraxacum* (*p* = 0.017), *Agrimonia* vs. *Taraxacum* (*p* = 0.001), and *Agrimonia* vs. *Cichorium* (*p* = 0.004) (Supplementary Materials Table S3).

The boxplot diagram showing the distribution and spread of the DPPH data set for every vegetal extract group is presented in Supplementary Materials Figure S16.

For the ABTS method, a test was conducted to determine whether the difference between the groups occurs randomly or is statistically significant, and it was observed that there are statistically significant differences (*p* < 0.05; Table 12) when comparing the antioxidant capacities of the studied plant extracts (One-Way ANOVA).

Thus, after performing the Games–Howell post hoc test for unequal variances, it was found that there was a statistically significant difference between: *Cynara* vs. *Agrimonia* (*p* = 0.017), *Cynara* vs. *Taraxacum* (*p* = 0.032), *Rosmarinus* vs. *Agrimonia* (*p* = 0.041), *Rosmarinus* vs. *Taraxacum* (*p* = 0.009), *Agrimonia* vs. *Taraxacum* (*p* = 0.001), and *Agrimonia* vs. *Cichorium* (*p* = 0.003). Table S4 from the Supplementary Materials details the *p* values for each comparison between the analyzed groups, as well as the degree of significance.

The boxplot diagram showing the distribution and spread of the ABTS data set for every vegetal extract group is presented in Supplementary Materials Figure S17.

For the FRAP method, the Tukey HSD post hoc test (for equal variances) showed that there are statistically significant differences between the analyzed extracts (*p* < 0.05; Table 13), in terms of obtained optical density.

Thus, the differences between groups are quantified in Supplementary Materials Table S5: *Cynara* vs. *Rosmarinus* (*p* = 0.000), *Cynara* vs. *Agrimonia* (*p* = 0.000), *Cynara* vs. *Cichorium* (*p* = 0.042), *Rosmarinus* vs. *Taraxacum* (*p* = 0.001), *Rosmarinus* vs. *Cichorium* (*p* = 0.020), *Agrimonia* vs. *Taraxacum* (*p* = 0.001), and *Agrimonia* vs. *Cichorium* (*p* = 0.017).

The boxplot diagram showing the distribution and spread of FRAP data set for every vegetal extract group is presented in Supplementary Materials Figure S18.

After calculating the Pearson coefficient (according to Supplementary Materials Table S6), a very strong correlation can be observed between the total phenolic content (TP) and the antioxidant action quantified by the IC50 values of the extracts, analyzed by the three methods (TP vs. DPPH: r = −0.956, *p* = 0.011; TP vs. ABTS: r = −0.930, *p* = 0.022; TP vs. FRAP: r = −0.979, *p* = 0.004). The values of the Pearson coefficient are negative, which explains the inverse correlation between the data (the higher the number of polyphenols, the lower the IC50 value of the extracts; therefore, the stronger the antioxidant action).

The values of the coefficients of determination (R^2^) are very high (91.3936%, 86.4900%, 95.8441%), which shows the association between the analyzed data and emphasizes the strong link between the polyphenol content of the extracts and the anti-radical effect exerted by them in the human body (Table 14). Thus, at least 86% of the variation in the antioxidant effect of the studied extracts is explained by the quantified polyphenol concentration in the plant extracts, demonstrating a direct influence on the chelating capacity of free radicals.

The Pearson correlation analysis also indicates that there is a statistically strong and inverse correlation between the concentration of phenolcarboxylic acids and the antioxidant effect of the plant extracts; as the IC50 value is lower, the TPA concentration is higher, and the extract is a better antioxidant (negative Pearson coefficient).

These results explain the special antiradical action of the studied plant extracts, as well as their annihilation power against reactive oxygen species at the tissue and cellular levels (TPA vs. DPPH: r = −0.973, *p* = 0.005, TPA vs. ABTS: r = −0.980, *p* = 0.003, TPA vs. FRAP: r = −0.965, *p* = 0.008). The results of the Pearson correlation analysis are presented in Supplementary Materials Table S7. The determination coefficient R^2^ is mathematically related to the Pearson correlation coefficient (r) and shows the magnitude of the association between the antioxidant action of the extracts evaluated by the three methodologies and the total phenolic acid concentration for each plant extract (Table 15).

### 2.5. Molecular Docking

A total of 23 identified polyphenolic compounds were subjected to molecular docking simulations to predict their binding affinities and molecular interactions with potential targets involved in hepatoprotection. The docking protocol was successfully validated by redocking the positive controls into the active sites, with the redocked ligands showing only slight variations in pose conformations. The CYP2E1 inhibitor showed a binding energy of –7.776 kcal/mol and 0.1704 Å RMSD after superposition on the initial conformation. The TNF-α inhibitor exhibited a docking score of –8.867 kcal/mol and 0.1325 Å RMSD, while redocking the GPx4 allosteric activator yielded a binding energy of –6.978 kcal/mol and 0.2691 Å RMSD (Supplementary Materials Figure S19).

Docking results for the screened polyphenols are shown in Table 16. Out of the 23 docked compounds, 2 ligands did not fit into the active site of CYP2E1, and the binding affinity could not be determined due to their large molecular volume (naringin and rutin). The binding energies after docking on CYP2E1 ranged from –8.942 kcal/mol to –0.884 kcal/mol, with a mean value of –7.128 ± 1.855 kcal/mol. Pinocembrin showed the highest binding affinity, while hyperoside exhibited the lowest affinity for CYP2E1. The highest ligand efficiency was observed for cinnamic acid (0.7417, –8.159 kcal/mol). Ten polyphenolic compounds showed higher affinities than the positive control (pinocembrin, chrysin, apigenin, formononetin, epicatechin, naringenin, catechin, cinnamic acid, *p*-coumaric acid, and abscisic acid).

The binding energies for TNF-α ranged from –8.998 to –5.673 kcal/mol, with a mean value of –7.382 ± 0.998 kcal/mol. The lowest binding energy was observed for rutin, and the highest energy for syringic acid. Moreover, cinnamic acid showed the highest ligand efficacy for TNF-α as well (0.5436, –5.980 kcal/mol). Only one screened compound exhibited higher binding affinities than the positive control (rutin), while three polyphenols showed slightly lower but comparable affinities (genistin, hyperoside, and naringin).

The molecular docking experiment targeted at the GPx4 allosteric binding site yielded binding energies ranging between –7.023 and –4.637 kcal/mol (–6.198 ± 0.789 kcal/mol). Naringin showed the highest affinity for the GPx4 allosteric site, while syringic acid showed the lowest affinity. Only two ligands had binding energies lower than the positive control (naringin and genistin), while six other compounds had similar predicted activity values (apigenin, rutin, chlorogenic acid, hyperoside, epicatechin, and kaempferol).

**Figure 8:**
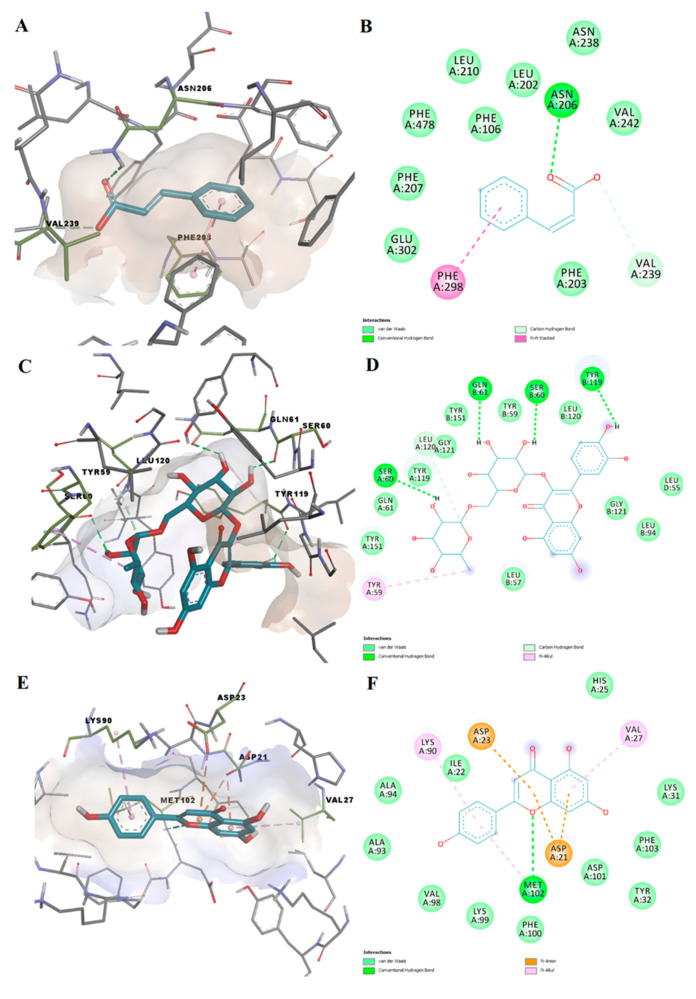
Docking poses and molecular interactions between docked ligands and target proteins. (**A**) 3D conformation of predicted cinnamic acid-CYP2E1 complex; (**B**) 2D depiction of protein–ligand interactions for predicted cinnamic acid–CYP2E1 complex; (**C**) 3D conformation of predicted rutin–TNF-α complex; (**D**) 2D depiction of protein–ligand interactions for predicted rutin–TNF-α complex; (**E**) 3D conformation of predicted apigenin–GPx4 complex; (**F**) 2D depiction of protein–ligand interactions for predicted apigenin–GPx4 complex.

The predicted molecular interactions are further discussed for the top scoring phytochemicals on each target. We chose to comment on the interactions between CYP2E1 and cinnamic acid since this particular compound showed a strikingly high ligand efficiency. Cinnamic acid is involved in hydrogen bonding with Asn206 through its carboxylic moiety. The protein–ligand complex is further stabilized by a carbon–hydrogen bond with Val239, pi–pi stacked interactions with Phe298, and van der Waals interactions with nine other residues within the active site (Figure 8A,B).

Rutin showed the highest predicted binding affinity for the TNF-α binding site. Rutin acted as a hydrogen bond donor for four residues (Ser60, Gln61, Tyr119) through several hydroxyl groups and formed a carbon–hydrogen bond with Leu120 (Figure 8C,D). Moreover, nonpolar interactions such as pi–alkyl (Tyr59) and van der Waals interactions were also responsible for binding to the active site. Apigenin had the third highest binding affinity for GPx4 among the screened phytochemicals, also showing a good ligand efficiency value. The binding potential of apigenin onto the GPx4 allosteric binding site is supported by a hydrogen bond with Met102, 2 pi–anion interactions with Asp21 and Asp23, 2 pi–alkyl interactions with Val27 and Lyes90, and 11 van der Waals interactions (Figure 8E,F).

## 3. Discussion

In the present paper, five types of plant extracts were analyzed: *Cynarae extractum*, *Rosmarini extractum*, *Taraxaci extractum*, *Cichorii extractum*, and *Agrimoniae extractum*. The qualitative chemical profile (UHPLC–HRMS/MS method) and quantitative analysis (spectrophotometric and UHPLC–HRMS/MS methods) were established. The antioxidant activity was determined in the acellular system (DPPH, ABTS, and FRAP), and docking studies were performed to predict the potential hepatoprotective action induced by the extracts. The chemical profile is dependent on the type of plant raw material from which the extracts were obtained, so the most ennobled extract in polyphenolic compounds was *Agrimoniae extractum*.

Qualitative and quantitative chemical analyses were used for the integrated characterization and comparative analysis of the polyphenolic profile and in vitro antioxidant action.

The polyphenol content in plant extracts is directly correlated with their antioxidant action. The large variety of chemical compounds identified in the study by the UHPLC–HRMS/MS technique emphasizes the need to evaluate the antioxidant action of plant extracts using at least three different methods of determination for the comparative analysis to be accurate.

Several phenolic compounds with high antioxidant activity were identified in the vegetal extracts using the UHPLC–HRMS/MS method. Most of them, mainly phenolic acids such as chlororgenic acid, *p*-coumaric acid, and ferulic acid, were identified in all the analyzed extracts.

A Hierarchical Cluster Analysis (HCA) was performed as a statistical technique that identifies groups of samples that behave similarly or show similar characteristics and thus quantifies the structural characteristics of the samples or variables. The procedure of hierarchical clustering involves the construction of a hierarchy of treelike structures. As it uses a hierarchical configuration—a tree called a dendrogram—to structure the data, cluster analysis is a quantitative form of classification in which classes are subclassified into groups [48,49].

We implemented the hierarchical clustering approach to identify, in each extract, the groups of identified compounds in relation to the response variables: exact mass, adduct ion (*m*/*z*)/monitored negative ion, and retention times.

The cluster analysis performed on the five plant extracts showed a heterogeneous distribution in a variable number of clusters for each type of extract; the results obtained were in direct correlation with the compounds identified by the UHPLC–HRMS/MS method. Thus, in CE, the 28 identified compounds were distributed into four groups; in RE, TE, and AE, although a variable number of compounds were identified, they were distributed into five groups each; and in CHE, where 43 compounds were identified, they were distributed into six clusters. The distributed compounds belong to the class of flavonoids, isoflavones, depsides, diterpenes, triterpenes, sesquiterpenes, phenolic acids, proanthocyanidins, and coumarin derivatives.

Furthermore, the comparative analysis of the antioxidant capacity of some vegetal extracts was assessed. Different antiradical activities were obtained for each extract due to the different types and content of their polyphenolic compounds and other phytochemical constituents. According to the results, the order of IC50 values in this assay was AE < RE < CHE < TE < CE. Thus, considering that a lower IC50 value signifies a higher total antioxidant activity, agrimony extract was the most potent antioxidant of all, followed by rosemary, chicory, dandelion, and artichoke.

The statistical analysis performed on the data sets, but also the statistically significant *p* values (*p* < 0.05) for the antioxidant results obtained by dispersion analysis within the same method of determination, support the obvious differences between the antiradical effects of the studied extracts.

Moreover, after calculating the Pearson coefficient (denoted r) and the coefficient of determination (denoted R^2^), a very strong correlation can be demonstrated not only between different antioxidant methodologies but also between the active principles and total antioxidant activity of the extracts. The obtained results for the phenolcarboxylic acids are very close to the ideal scientific value of 100% (R^2^ = 94.6729%, R^2^ = 96.0400%, R^2^ = 93.1225%). This confirms the existence of a relevant association between the data, and emphasizes, together with the Pearson correlation analysis, the interdependence of the data sets and the conclusion of this study. Although the two coefficients are calculated differently, they describe the relationship between data sets and measure the magnitude of the relationship between the analyzed data. In all determinations, the Pearson correlation coefficient values are greater than 0.900, which reveals the highest correlation between the antioxidant activity of the extracts determined using three different methodologies and analyzed by pairs of data. As the coefficient of determination expressed as a percentage can have a major impact and can also be more informative (intuitively) than other statistical measures, it is more robust for poor data set matches and is therefore more accurate.

The antioxidant activity of the analyzed extracts, associated with a high polyphenolic content, could explain their hepatoprotective potential. Some important elements link hepatotoxicity and oxidative stress, particularly the production of reactive oxygen species and other reactive species, which can damage hepatocyte integrity and functionality and contribute to the pathogenesis of many liver diseases.

The antioxidant activity and hepatoprotective potential for the studied herbal products and extracts were confirmed in other studies as well. In a clinical study reported in the literature, the benefits of artichoke extract supplementation were shown in patients with metabolic syndrome, where a significant decrease in LDL cholesterol was observed, based on the antioxidant effects induced by polyphenolic compounds [50]. In addition, an important decrease in malondialdehyde (MDA) levels in the liver was observed in preclinical studies [51].

According to the studies of Lombardo et al. (2010) [52], and Nouraei et al. (2018) [53], the concentration of polyphenols in Cynara scolymus is influenced by the harvest time, species genotype, soil, and climatic conditions [12]. According to the studies reported by Allahdadi M., and Farzaneh P. in 2018, the use of soil fertilizers leads to a decrease in the content of TPA, TF, and TP in artichokes [54]. We assume that the low concentration of total polyphenols in the extract obtained in our study is due to an inadequate collection of the plant product. These statements are based on the results obtained during the evaluation of the plant’s raw materials.

The compounds quantified in CE plant extracts fit within the profile of polyphenols cited in the literature [55,56]. The identification of pinocembrin in large quantities (43.1 µg/g) in RE is consistent with the literature data, as this compound was also identified in rosemary honey [57,58]. The chemical profile of the compounds identified in TE is also consistent with studies reported in the literature [59,60].

According to Gordon’s studies [61], the polyphenolic derivatives of rosemary extract act as primary antioxidants, annihilating lipid and hydroxyl radicals. Fang et al. [62] consider that they may act as chelating agents, especially on metal ions such as Fe^2+^, thereby reducing the generation of free radicals.

Carnosic acid and carnosol act as potent peroxyl radical scavengers since they are strong inhibitors of lipid peroxidation in liposomal and microsomal systems. Thus, carnosic acid specifically scavenges H_2_O_2_ and can also act as a substrate for peroxidase. Additionally, rosmanol has been reported to induce an antioxidant effect four times higher than various synthetic compounds used in tests as a reference for its antioxidative effectiveness (BHT-butylhydroxytoluene, BHA-butylhydroxyanisole) [22].

Ivanov [63] shows that the presence of sinapic and chicoric acid, correlated with the high number of polyphenols, justifies the good antioxidant effect of the dandelion extract. Atef et al. [64] highlighted the role that an aqueous extract obtained from chicory played in increasing the level of reduced glutathione (GSH) in damaged liver tissue, following CCl_4_ poisoning in experimental animal models. According to the studies of Correia et al. [65], Ivanova et al. [40], and Santos et al. [66], *Agrimonia extracts* have a significant capacity to capture reactive oxygen species (ROS), an effect which is correlated with an increased polyphenol content.

A previous study [67] regarding the hepatoprotective and radical scavenging activity of rosemary on alcoholic liver disease reported the remarkable capacity of *Rosmarinus officinalis* to diminish the level of serum hepatic enzymes such as ALT (alanine aminotransferase) and ACP (acid phosphatase). The hepatoprotective effect in the rat model of alcoholic liver disease is due to its mechanism of action and the antioxidant effect, which leads to the reduction of liver tissue damage.

According to other scientific studies, dandelion showed a significant hepatoprotective action in animal studies, which was validated by the reduction of all the determined liver markers. These findings support its effective antioxidant activity in toxin-induced oxidative stress or damage [68].

In addition, the administration of chicory extract in experimental rats demonstrated hepatoprotective and antihepatotoxic effects against dexamethasone-induced alterations. These effects are due to the content of polyphenols and other antioxidants that can reduce the activity of liver enzymes [69].

Furthermore, the superior antioxidant activity of agrimony over artichoke and other vegetal extracts is stated in Kuczmannová et al.’s work [70]. This article suggests a relationship between the hepatoprotective effects and the antioxidant properties induced by polyphenols, which are the dominant compounds in the extract.

Free radical scavenging activity is by no means the sole molecular mechanism responsible for the hepatoprotective effects of some phytochemicals. The hepatoprotection exerted by plant extracts can be achieved by the interaction of phytoconstituents with biological targets that either promote or alleviate toxicity at the hepatocellular level. For instance, inhibition of CYP2E1 activity impairs the conversion of xenobiotics and polyunsaturated fatty acids to toxic metabolites that can promote liver damage [45]. Inflammation and oxidative stress can be alleviated by down-regulating the expression and activity levels of cytokines (TNF-α, interleukins) or by up-regulating key enzymes with antioxidant and anti-inflammatory roles, such as GPx4 [46,47]. Moreover, gomisin A, a bioactive compound found in *Schisandra chinensis*, was reported to ameliorate carbon tetrachloride-induced hepatotoxicity in rats by inhibiting the activation of nuclear factor kappa B (NF-κB), down-regulating inducible nitric oxide synthase (iNOS) expression, and reducing fibrogenesis [71].

The hepatoprotective potential of the studied herbal extracts can be supported not only by the aforementioned findings but also by our molecular docking results. Some of the detected polyphenols exhibited satisfying predicted binding affinities and ligand efficiencies for the selected biological targets (CYP2E1, TNF-α, and GPx4). Therefore, the phytochemical constituents have the potential to exert hepatoprotective effects by either inhibiting cytochrome P450 2E1 isoform and tumor necrosis factor alpha or activating glutathione peroxidase 4 through an allosteric mechanism. The docking results of the screened phytochemicals can also be correlated with previously published studies. For instance, chrysin, catechin, and apigenin have been shown to inhibit CYP2E1 activity and improve redox balance, while cinnamic acid and syringic acid have been reported to ameliorate hepatotoxicity in laboratory animal studies [72,73]. Other studies revealed that catechin, hyperoside, rutin, genistein, and naringin suppress TNF-α mediated inflammatory responses in various in vitro and in vivo experiments [74,75,76,77,78]. Furthermore, another study highlighted that apigenin activates glutathione peroxidase 4 and alleviates ferroptosis and oxidative stress, which is in concordance with our molecular docking results [79]. Furthermore, it has been shown that naringenin alleviates myocardial ischemia/reperfusion injury by regulating the Nrf2/System xc-/GPx4 axis [80], while rutin induces glutathione peroxidase activity in cadmium-induced oxidative stress [81].

Following the in vitro determination of the antioxidant effect of the studied extracts, the extracts’ in vivo action against oxidative stress at the cellular and tissue level can be quantified in future research.

## 4. Materials and Methods

### 4.1. Formulation of Vegetal Extracts

#### 4.1.1. Plant Materials, Reagents, and Equipment

Herbal products were purchased as one-component medicinal teas from specialized pharmaceutical units in Romania; the products comprise: artichoke leaves—*Cynarae folium* (CF), rosemary leaves—*Rosmarini folium* (RF), dandelion aerial parts—*Taraxaci herba* (TH), common chicory aerial parts—*Cichorii herba* (CH), and agrimony aerial parts—*Agrimoniae herba* (AH).

Based on previously reported studies [82], the solvents used for the extraction were 50% ethanol for CF, RF, CH, and AH, and 20% ethanol for TH. The choice of the solvent was motivated by the need to obtain the optimal extraction of phenolic compounds for all analyzed herbal products, and 20% ethanol was the best extraction solvent for dandelion aerial parts (the highest extraction yield for TF and TPA). The ethanol used as solvent in this section was purchased from Sigma-Aldrich, Darmstadt, Germany.

Aliquots of 50 g of each herbal product were subjected to two consecutive reflux extraction processes: the first one, using 1.5 L of solvent for 30 min, and the second one, using 750 mL of solvent, also for 30 min. The two extract solutions were mixed and concentrated in a rotary evaporator (Buchi, Vacuum Pump V-700) and then subjected to a lyophilization process (Christ Alpha 1-2/B Braun, BiotechInt). The dry extracts were conserved in a glass *vacuum desiccator*. The samples were marked as follows: CE (*Cynarae extractum*), RE (*Rosmarini extractum*), TE (*Taraxaci extractum*), CHE (*Cichorii extractum*), and AE (*Agrimoniae extractum*).

Other equipment and experimental conditions used in this study are presented in each stage of the experiments.

#### 4.1.2. Determination of the Quality of Plant Extracts

Spectrophotometric methods were used for the determination of total phenolic content (TP), total flavonoid content (TF), total phenolic acid content (TPA), and in vitro antioxidant activity (AA). The polyphenolic profile of the vegetal extracts was established based on non-targeted tandem mass spectrometry (MS-MS) using the hyphenated technique represented by Ultra-High Performance Liquid Chromatography (UHPLC) coupled with the Q-Exactive High Resolution Mass Spectrometer (HRMS). The same method was used for the quantification of selected polyphenolic compounds for each available analytical standard (Sigma-Aldrich, Germany).

1. Determination of total flavonoid content (TF)

A colorimetric method based on the reaction of flavonoids and AlCl_3_ was used for the total flavonoid content assay. Aliquots of 0.2 g extract were dissolved in 25 mL of 50% ethanol for CE, RE, AE, and CHE, or 20% ethanol for TE, depending on the solvent used for the formulation of each dry extract. Volumes of 0.4 mL, 0.6 mL, 0.8 mL, 1 mL, and 1.2 mL were poured into 10 mL volumetric flasks. Then, 2 mL of sodium acetate 100 g/L (Sigma-Aldrich, Germany) and 1 mL of aluminum chloride solution 25 g/L (Sigma-Aldrich, Germany) were added. Further, all the volumes were adjusted to 10 mL by adding the same solvent as above. In parallel with the samples to be analyzed, the appropriate control samples were prepared under the same conditions but lacked sodium acetate and aluminum chloride. After 45 min, the absorbance was measured at 427 nm (Jasco V-530 spectrophotometer, Tokyo, Japan). Rutin (Sigma-Aldrich, Germany) was used as a standard for the linear calibration curve in the concentration range of 5–35 μg/mL with R^2^ = 0.9992 (Supplementary Materials Figure S20). The total flavonoid content (TF) of the extract was expressed as mg rutin equivalents per gram of sample (mg/g) [83].

2. Determination of total phenolic acid content (TPA)

Total phenolic acid content was measured based on its property of forming nitro derivatives with nitrous acids. For CE, TE, and CHE, equal amounts of 0.2 g dry extracts were dissolved in 25 mL of 50% ethanol (for CE and CHE) and 20% ethanol (for TE), depending on the solvent used for the formulation of each dry extract. For RE and AE, 0.1 g of dry extract were dissolved in 100 mL of 50% ethanol. We used different solvents and quantities of dry extract according to our previous published results to provide each analyzed extract with the extraction with the highest concentration of target compounds.

Volumes of 0.8 mL, 1 mL, 1.2 mL, 1.4 mL, and 1.6 mL were poured into 10 mL volumetric flasks. Then, 2 mL of hydrochloric acid 0.5 M (Sigma-Aldrich, Germany), 2 mL of Arnow reagent (Sigma-Aldrich, Germany), and 2 mL of sodium hydroxide 85 g/L (Sigma-Aldrich, Germany) were successively added. After this, all the volumes were adjusted to 10 mL by adding distilled water. The absorbance was immediately measured at 525 nm (Jasco spectrophotometer, Japan), and compared to a sample that lacks the Arnow reagent. Chlorogenic acid (Sigma-Aldrich, Germany) was used as a standard for the calibration curve in the linear range of 11–53 μg/mL with R^2^ = 0.9998. (Supplementary Materials Figure S21). The total phenolic acid content (TPA) of the extract was expressed as mg chlorogenic acid equivalents per gram of sample (mg/g) [83].

3. Determination of total phenolic content (TP)

The determination of total polyphenols (TP) was performed according to Lamuela-Raventós [84] with slight modifications. Aliquots of 0.1 g dry extract were dissolved in 100 mL of 50% ethanol for CE, RE, AE, and CHE, or 20% ethanol for TE, depending on the solvent used for the formulation of each dry extract. Volumes of 0.5 mL, 0.6 mL, 0.7 mL, 0.8 mL, and 0.9 mL were poured into 10 mL volumetric flasks and adjusted to 1 mL by adding distilled water. Then, the volumes were mixed with 1 mL of Folin–Ciocalteu’s phenol reagent (Sigma-Aldrich, Germany) and kept at 25 °C for 5–8 min before adding 8 mL of sodium carbonate solution 200 g/L (Sigma-Aldrich, Germany). After 40 min in dark conditions, the absorbance was measured at 725 nm (Jasco V-530 spectrophotometer, Japan). The absorbance was measured relative to a blank sample obtained by mixing 1 mL of distilled water with 1 mL of Folin–Ciocalteu’s reagent and then adjusted to 10 mL by adding sodium carbonate. Tannic acid (Sigma-Aldrich, Germany) was used as a standard for the calibration curve in a linear concentration range of 2–9 µg/mL with R^2^ = 0.999 (Supplementary Materials Figure S22). The total phenolic content (TP) was expressed as mg tannic acid equivalents per gram of sample (mg/g).

4. Identification and quantification of polyphenolic compounds by Ultra-High Performance Liquid Chromatography coupled with High Resolution Mass Spectrometry (UHPLC–HRMS/MS)

Analytical standards of 30 compounds (8 phenolic acids, 7 isoflavones, and 15 flavonoids) were purchased from Sigma-Aldrich, Germany. Methanol and ethyl alcohol, HPLC grade, were purchased from Merck Romania; formic acid (98%) and ultrapure water (LC-MS grade) were also purchased from Merck (Merck Romania, Romania). For the calibration of the Orbitrap Mass Spectrometer, the Pierce LTQ Velos ESI positive and negative ion calibration solutions (Thermo Fisher Scientific, Germany) were used.

Although ethanol was used in the preparation of plant extracts, methanol was used for the preparation of standard solutions considering the higher stability of the methanolic solution. However, ethanol or methanol used as a solvent does not influence the quantitative results in mass spectrometry.

Individual stock standard solutions with a concentration of 1.0 mg/mL in methanol were prepared for each compound. A series of mixed-working standard solutions (concentration ranged from 0.05 to 1.0 µg/mL) were prepared by successive dilution of the mixture of standard solutions with 20% methanol. All the solutions were stored at −20 °C before use.

- **LC parameters**

The analysis was carried out using a Thermo Scientific Dionex Ultimate 3000 UHPLC system consisting of a pump (Series RS) coupled with a column compartment (Series TCC-3000RS) and an autosampler (Series WPS-3000RS). UHPLC system was controlled by Chromeleon 7.2 Software (Thermo Fisher Scientific, Waltham, MA and Dionex Softron GMbH Part of Thermo Fisher Scientific, Germany).

A 35-min gradient over an ultra-performance Accucore UHPLC Column C18 (150 × 2.1 mm, 2.6 µm), (Thermo Fisher Scientific, Germany) was applied. The column temperature was set to 40 °C. The mobile phase consisted of eluent A, ultrapure water containing 500 µL/L formic acid (pH 2.5) and eluent B, and methanol with 500 µL/L formic acid. The step gradient was as follows: 0–1 min 100% A; 1.0–10.0 min linear increase to 30% B; 10.0–26.0 min linear increased to 100% B, and held for 4.0 min; 30.0–32.5 min decreasing to 0% B; equilibration time of 2.5 min. The run was performed at 0.3 mL/min for a total of 35 min.

- **MS parameters**

A HESI (Heated ElectroSpray Ionization) ion source was used for the ionization in the negative mode. The ion source parameters were optimized as follows: the nitrogen as sheath and the auxiliary gas flow rate were set to 8 and 6 units, respectively. The source heater temperature was set to 300 °C, the capillary temperature was set to 300 °C, the auxiliary gas heater temperature was set to 300 °C, the electrospray voltage was set to 2800 V, and the S-lens RF level was set to 50.

The full-scan HRMS analysis of the compounds was performed using a Q-Exactive Mass Spectrometer. Full-scan data in negative mode were acquired at a resolving power of 70,000 FWHM at *m*/*z* 200. A scan range of *m*/*z* 100–1000 Da was chosen; the Automatic Gain Control (AGC) was set at 3 × 10^6^ and the injection time was set to 200 ms. Scan-rate was set at 2 scan/s. External calibration was performed by the calibration solution in positive and negative mode.

For structural information, a vDIA (variable Data Independent Acquisition) approach was selected for untargeted HRMS/MS analysis. A total of six scan events were combined: one full scan event with the mentioned parameters and five MS-MS events. In the MS2 scan events, the precursor ion ranges from *m*/*z* 95–205, 195–305, 295–405, 395–505, and 500–10,005 were consecutively selected, fragmented in the HCD cell, and measured in five separate Orbitrap scans at a resolving power of 35,000 FWHM. The fragmentation events were performed at 30, 60, and 80 NCE (Normalized Collision Energy). The C-trap parameters for all scan events were as follows: the Automatic Gain Control (AGC) target was 1 × 10^6^ and the injection time was 100 ms.

Data were processed by the Quan/Qual Browser Xcalibur 2.3 (Thermo Fisher Scientific, Germany). The mass tolerance window was set to 5 ppm.

Validation parameters of the LC-HRMS analytical method are presented in Supplementary Materials Table S8.

In MS-MS analysis, detection of at least two fragment ions was performed by comparing them to the reference standards. For those compounds without available references, the structures were presumed based on high-accuracy analysis of deprotonated precursors and fragment ions of specific components. The chemical elemental composition for each target peak was assigned within a mass error of 2 ppm using the chemical Chemspider database (www.chemspider.com (accessed on 15 March 2022)). Based on the literature [85,86,87,88,89], a self-built chemical database of polyphenolic compounds that were known to be in the selected plants was achieved. The fragment ions from MS-MS analysis were used to further confirm the chemical structure by comparing the analysis results with MS-MS data from NORMAN MassBank (https://massbank.eu/MassBank/ (accessed on 15 March 2022)), mzCloude Advanced Mass Spectral Database (https://www.mzcloud.org/ (accessed on 15 March 2022)), and PubChem (https://pubchem.ncbi.nlm.nih.gov/ (accessed on 4 April 2022)). For a comparison analysis, ACDLabs MS Fragmenter 2019.2.1 software was used to generate the fragmentation pattern of the identified compounds (Supplementary Materials Table S9).

The optimization of the UHPLC and MS conditions and the validation of the quantitative method are presented in the Supplementary Materials.

### 4.2. Determination of Antioxidant Activity (AA)

#### 4.2.1. DPPH Free Radical Scavenging Activity

The free radical scavenging activity of the plant extracts was measured by using the diphenyl-picrylhydrazyl (DPPH) assay, according to Çelik S.E., where the antioxidant activity is influenced by both the characteristics of the substrate and the polarity of the solvent [90]. Equal amounts of 0.25 g dry extracts were dissolved in 25 mL of 50% ethanol for CE, RE, AE, and CHE, and 20% ethanol for TE, depending on the solvent used for the formulation of each dry extract. Volumes of 10 µL, 20 µL, 30 µL, 40 µL, 50 µL, 60 µL, 70 µL, 80 µL, 90 µL, and 100 µL of each obtained solution were poured into 10 mL volumetric flasks and adjusted to 10 mL by adding the same solvent as above. An amount of 0.5 mL of each dilute solution was mixed with 3 mL of 0.1 mM DPPH radical solution (Sigma-Aldrich, Germany) [91]. The solutions were held in the dark for 30 min, and the absorbance was then measured at 515 nm using a spectrophotometer (Jasco, Japan). Ascorbic acid (Sigma-Aldrich, Germany) was used as a reference for the calibration curve in the range of concentration between 2–22 µg/mL (Supplementary Materials Figures S23 and S24).

The percentage of inhibition of DPPH^•^ was calculated using the formula below:

(1) $$\;\%\;Inhibition_{DPPH}=\frac{A\;\left(blank\right)-A\;\left(sample\right)}{A\;\left(blank\right)}\times 100,$$

ref. [92], where: 

A (blank) = blank absorbance of 0.1 mM DPPH solution in the absence of extracts (1.00 ± 0.10);

A (sample) = sample absorbance of the DPPH solution in the presence of extracts after 30 min.

Based on the established values, inhibition curves (%) were constructed depending on the concentration (mg/mL). Using the linear equations, the IC50 values (mg/mL) were determined for each extract (for the value y = 50).

#### 4.2.2. ABTS Method of Total Antioxidant Capacity Assessment

The ABTS assay is considered one of the most sensitive techniques to measure the antioxidant activity of both hydrophilic and lipophilic compounds due to the response of antioxidants involving faster reaction kinetics in a pH-independent manner [93,94].

The ABTS radical cation (ABTS^•+^) was obtained by reacting the ABTS (Sigma-Aldrich, Germany) 7.4 mM solution with 2.6 mM potassium persulfate (K_2_S_2_O_8_-Sigma-Aldrich, Germany) and keeping the mixture in the dark at room temperature for 16 h before use [95].

Equal amounts of 0.25 g dry extracts were dissolved in 25 mL of 50% ethanol for CE, RE, AE, and CHE, or 20% ethanol for TE, depending on the solvent used for the formulation of each dry extract. Volumes of 10 µL, 20 µL, 30 µL, 40 µL, 50 µL, 60 µL, 70 µL, 80 µL, 90 µL, and 100 µL of each obtained solution were poured into volumetric flasks and adjusted to 10 mL by adding the same solvent as above. An amount of 0.5 mL of each dilute solution was mixed with 3 mL of ABTS^•+^ solution diluted with ethanol (Sigma-Aldrich, Germany). The solutions were stirred and held in the dark for 6 min [96]. The absorbance was then measured at 734 nm, relative to absolute ethanol, using a spectrophotometer (Jasco, Tokyo, Japan).

The percentage of inhibition of ABTS^•+^ was calculated using the formula below:

(2) $$\;\%\;Inhibition{\;}_{ABTS}=\frac{A\;(t=0\min)-A\;(t=6\min)}{A\;\left(t=0\;\min\right)}\times 100{,}_{\;}\mathrm{where}:$$

A (t=0 min) = absorbance of the blank sample (ABTS^•+^ sol in the absence of tested compounds: 0.70 ± 0.02);

A (t=6 min) = absorbance of the vegetal extract (ABTS^•+^ sol in the presence of tested compounds).

IC50 value, the concentration of sample required to scavenge 50% of the ABTS^•+^ free radical, was calculated from the plotted graph of radical scavenging activity against the concentration of extracts (IC–*inhibition*). The lower the IC50 value for an extract, the stronger the antioxidant activity.

#### 4.2.3. Antioxidant Activity Using FRAP Assay (Ferric Reducing Antioxidant Power Assay)

The ferric reducing power of plant extracts was determined using a modified FRAP assay [94]. The assay measures the antioxidant potential through the reduction of ferric iron (Fe^3+^) to ferrous iron (Fe^2+^) by antioxidants present in the samples. Following the reduction of ferric iron (Fe^3+^) to ferrous iron (Fe^2+^), a blue color develops.

Equal amounts of 0.25 g dry extracts were dissolved in 25 mL of 50% ethanol for CE, RE, AE, and CHE, or 20% ethanol for TE, depending on the solvent used for the formulation of each dry extract. Volumes of 50 µL, 60 µL, 70 µL, 80 µL, 90 µL, 100 µL, 200 µL, 300 µL, 400 µL, and 500 µL of each obtained solution were poured into volumetric flasks and adjusted to 10 mL by adding the same solvent as above. An amount of 2.5 mL of each dilute solution was mixed with phosphate buffer pH 6.6 (Sigma-Aldrich, Germany) and 2.5 mL of K_3_(FeCN)_6_ 1% (Sigma-Aldrich, Germany) before being heated to 50 °C for 20 min. Then, 2.5 mL of trichloroacetic acid (Sigma-Aldrich, Germany) was added to each sample. To 2.5 mL of each of the resulting solutions, 2.5 mL of distilled water and 0.5 mL of 0.1% FeCl_3_ (Sigma-Aldrich, Germany) were added, then left to stand for 10 min. The change in the absorbance at 700 nm was measured relative to a blank sample obtained by mixing 5 mL of distilled water with 0.5 mL of FeCl_3_ 0.1%.

The antioxidant capacity was determined using the EC50 value (mg/mL) as the concentration of the solutions at which the absorbance has a value of 0.5 (at half the antioxidant effect; EC—*effective*).

Due to the variability of plant properties and the nonuniformity of pharmacognostic profiles of vegetal extracts, different extract volumes were tested to reach the absorbance value of 0.5 (the more the experimental values obtained are around the point to be determined (EC50 for y = 0.5), the more accurate its approximation is). The optimized values have been set as above to perform a relevant comparative analysis within the same method and between different methods of assessing the antioxidant capacity.

### 4.3. Molecular Docking Simulations

A molecular docking experiment was carried out for several determined phytochemicals to evaluate the potential hepatoprotective effect of the plant extracts. The RCSB PDB database was used to retrieve the crystal structures of human CYP2E1 (PDB ID: 3LC4, 3.10 Å resolution) [97], TNF-α (PDB ID: 2AZ5, 2.10 Å resolution) [98], and GPx4 (PDB ID: 2OBI, 1.55 Å resolution) [99]. Protein structures were prepared for docking with YASARA Structure [100] by removal of solvent molecules and ions, correction of structural errors, protonation according to the physiological pH (7.4), and optimization of the hydrogen-bonding network. The structures of the retrieved protein–ligand complexes were thereafter minimized with NOVA2 forcefield. The co-crystallized ligands (omega-imidazolyl-dodecanoic acid for CYP2E1, and SPD304–6,7-dimethyl-3-[[methyl-[2-[methyl-[[1-[3-(trifluoromethyl)phenyl]indol-3-yl]methyl]amino]ethyl]amino]methyl]chrom-en-4-one for TNF-α) were removed and redocked into the active sites for validation of the docking protocol. Since the deposited GPx4 structures have no bound ligands, we retrieved a predicted complex with a known allosteric activator [101] (compound 1d4–1-{4-[(2-azaniumylethyl)sulfamoyl]phenyl}-3-cyclopentylthiourea) and redocked the ligand. The poses of redocked compounds were superposed on the initial conformation of the protein–ligand complexes to calculate the Root-Mean-Square Deviation (RMSD) values. The ligands used for validation also served as positive controls for docking score comparisons [102,103].

The SMILES codes of the selected phytochemicals for docking were retrieved from PubChem database. The ligand preparation protocol consisted of the generation of corresponding 3D structures with DataWarrior 5.2.1 [104], energy minimization using MMFF94s+ forcefield, and protonation at pH 7.4. Phytochemicals and positive controls were docked using AutoDock Vina v1.1.2 [105] algorithm within YASARA. The search space (25 × 25 × 25 Å) was centered around the co-crystallized ligands within the binding sites and 12 docking runs were performed for each ligand.

Docking results were retrieved as the binding energy or docking score (ΔG, kcal/mol) and ligand efficiency (LE, ΔG\no. of heavy atoms) of the best binding pose for each compound. The conformations of the predicted protein–ligand complexes and molecular interactions were analyzed using BIOVIA Discovery Studio Visualizer (BIOVIA, Discovery Studio Visualizer, Version 17.2.0, Dassault Systèmes, 2016, San Diego, CA, USA).

### 4.4. Statistical Analysis

All statistical analysis was performed using IBM SPSS Statistics software version 28.0 (IBM Corporation, Armonk, NY, USA). For each set of experimental data, the essential conditions for the application of statistical tests were evaluated, such as the normality of data and the homogeneity of variances. The normal distribution of the data was assessed by the Shapiro–Wilk test and by histograms. To detect significant differences between the data groups, One-Way ANOVA (Single-factor ANOVA) and Post Hoc Tests were applied for mean comparison: Tukey HSD for equal variances, or Games–Howell for unequal variances, depending on data distribution, sampling dispersion, and the number of studied groups. The Levene’s test was used to verify the homogeneity of variances for the experimental data sets. The Welch ANOVA analysis was run as a robust test when the condition of the homogeneity of variances was violated. When certain experimental data did not follow a Gaussian distribution, they were transformed (by logarithm in base 10), so that they became normally distributed and could be subjected to statistical tests. The correlation between certain groups of analyzed experimental data was also established by calculating the Pearson correlation coefficient. Interpretations were made after the mandatory application criteria were met (continuity of variables, independence of measurements, normality and linearity of data, and absence of outliers). In all cases, the significance level was set at 0.05. When *p* < 0.05, the obtained results were considered statistically significant.

Clustering analysis was also performed. The purpose of a clustering analysis is to construct groups or classes with two properties; within a group, observations behave similarly, and there must be dissimilarities between the observations of two distinct groups [106]. We implemented the hierarchical clustering approach to identify in each extract the homogeneous groups of compounds in relation to the response variables of exact mass, adduct ion (*m*/*z*)/monitored negative ion, and retention time. Since the variables have different units of measurement, we first performed a scaling and then calculated the distance based on the “average” method. The optimal number of groups was identified by the “silhouette” method.

## 5. Conclusions

In this study, the polyphenolic profile and in vitro antioxidant activity of *Cynarae extractum*, *Rosmarini extractum*, *Taraxaci extractum*, *Cichorii extractum*, and *Agrimoniae extractum* were characterized and compared. According to our results, all the vegetal extracts exert antioxidant properties due to their different content of phytochemicals, with agrimony extract being the most potent antioxidant of all. Nevertheless, due to their synergistic effect, all the studied extracts can be combined into herbal formulas for future applications in the field of phytomedicine to prevent hepatic disorders and protect human liver cells against oxidative damage and inflammation.

Future research will focus on determining the cytotoxicity of the studied vegetal extracts and performing preclinical tests on experimental animals to highlight the in vivo effects. Moreover, the impact on key biochemical parameters will be quantified by applying histological tests to liver tissue.

## Figures and Tables

**Figure 1:**
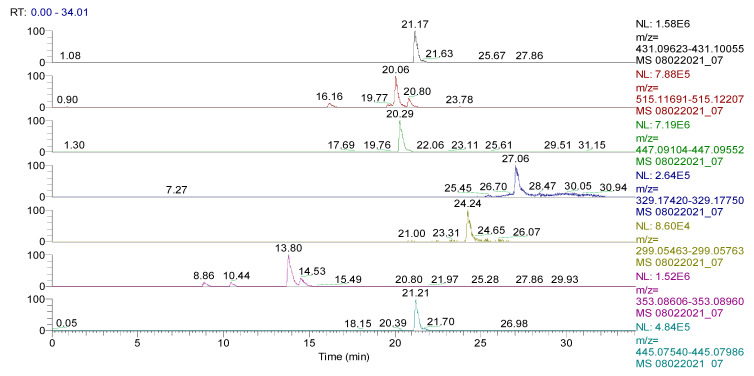
UHPLC–HRMS/MS chromatogram for **CE** in which were identified (top to bottom): vitexin (*m*/*z*: 431.09839, Rt: 21.17), 1,5-dicaffeoylquinic acid (*m*/*z*: 515.11949, Rt: 20.06), kaempherol (luteolin)-O-glucoside (*m*/*z*: 447.09331, Rt: 20.29), carnosol (*m*/*z*: 329.17585, Rt: 27.06), hispidulin (*m*/*z*: 299.05613, Rt: 24.24), chlorogenic/neochlorogenic acid (*m*/*z*: 353.08783, Rt: 10.44/13.80), and apigenin-7-O-glucuronide (*m*/*z*: 445.07763, Rt: 21.21).

**Figure 3:**
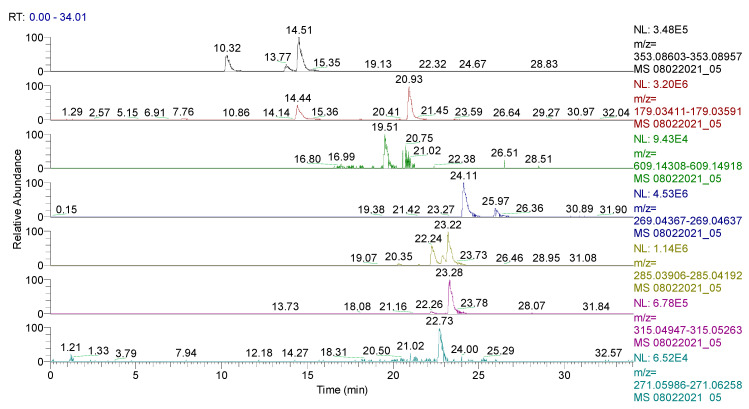
UHPLC–HRMS/MS chromatogram for **RE** in which were identified (top to bottom): chlorogenic acid (*m*/*z*: 353.08783, Rt: 10.32/14.51), caffeic acid/isomers (*m*/*z*: 179.03501, Rt: 14.44/20.93), rutin (*m*/*z*: 609.14613, Rt: 19.51), apigenin (*m*/*z*: 269.04502, Rt: 24.11), kaempferol (*m*/*z*: 285.04049, Rt: 23.22), naringenin (*m*/*z*: 271.06122, Rt: 22.73).

**Figure 4:**
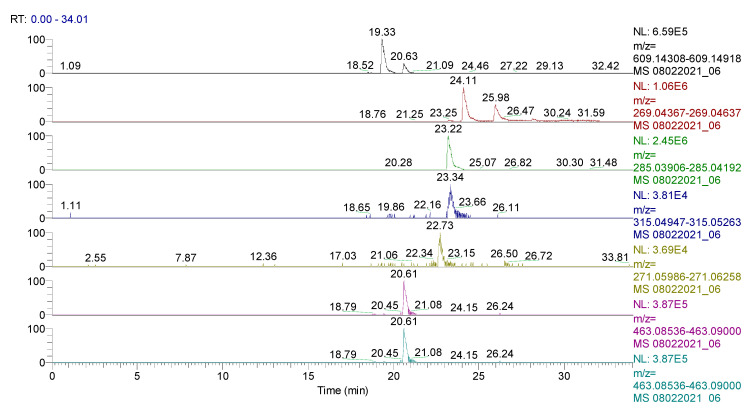
UHPLC–HRMS/MS chromatogram of **TE** in which were identified (top to bottom): rutin (*m*/*z*: 609.14613, Rt: 19.33), apigenin/genistein (*m*/*z*: 269.04502, Rt: 24.11), kaempherol (*m*/*z*: 285.04049, Rt: 23.22), naringenin (*m*/*z*: 271.06122, Rt: 22.73), and hyperoside (*m*/*z*: 463.08768, Rt: 20.61).

**Figure 5:**
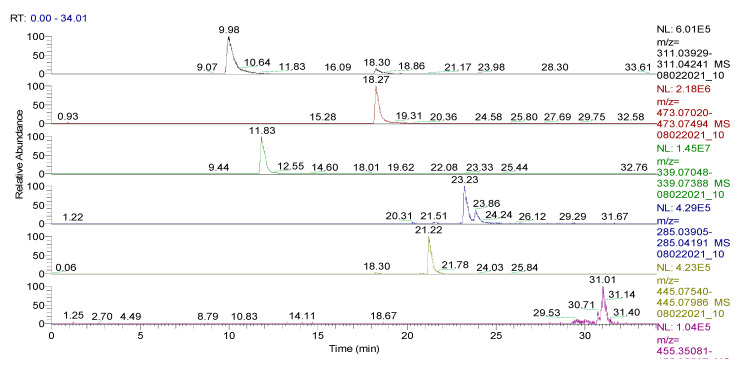
UHPLC–HRMS/MS chromatogram for **CHE** in which were identified (top to bottom): caftaric acid (*m*/*z*: 311.04085, Rt: 9.98), chicoric acid (*m*/*z*: 473.07257, Rt: 18.27), cichorin (*m*/*z*: 339.07218, Rt: 11.83), luteolin (*m*/*z*: 285.04048, Rt: 23.23/23.86), apigenin-7-O-glucuronide (*m*/*z*: 445.07763, Rt: 21.22), oleanolic acid (*m*/*z*: 455.35309, Rt: 31.01).

**Figure 7:**
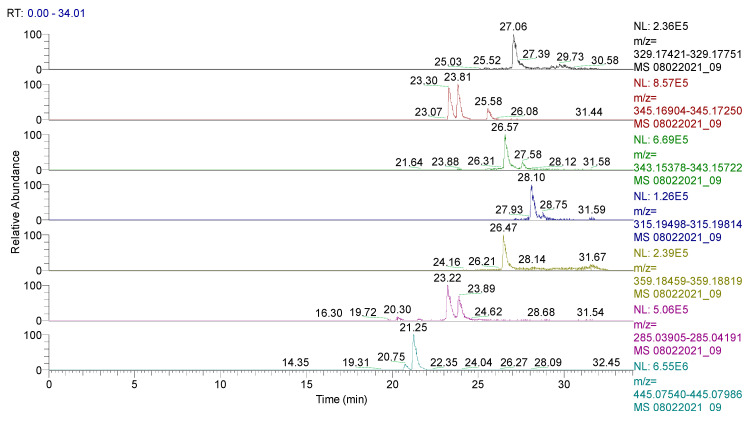
UHPLC–HRMS/MS chromatogram for **AE** in which were identified (top to bottom): carnosol (*m*/*z*: 329.17585, Rt: 27.06), rosmanol/epirosmanol (*m*/*z*: 345.17077, Rt: 23.07/23.81), rosmadial (*m*/*z*: 343.15509, Rt: 26.57), rosmaridiphenol (*m*/*z*: 315.19656, Rt: 28.10), rosmanol methyl ether (*m*/*z*: 359.18639, Rt: 26.47), luteolin (*m*/*z*: 285.04048, Rt: 23.22/23.89), and apigenin-7-O-glucuronide (*m*/*z*: 445.07763, Rt: 21.25).

**Table 1:** Quantitative analysis of active compounds in vegetal extracts.

Vegetal Extract	TPA (g Chlorogenic Acid/100 g Dry Extract)	TF (g Rutoside/100 g Dry Extract)	TP (g Tannic Acid/100 g Dry Extract)
CE	1.7389 ± 0.0904	2.2942 ± 0.1020	5.7627 ± 0.6946
RE	17.3293 ± 0.5010	6.0847 ± 0.3025	31.0913 ± 1.9781
TE	7.7644 ± 0.7846	1.9019 ± 0.1080	7.0016 ± 0.1686
CHE	7.7066 ± 0.7596	4.2714 ± 0.3628	16.1272 ± 0.6446
AE	24.1528 ± 1.1936	4.6713 ± 0.5440	31.7017 ± 1.2211

Total phenolic acid content (TPA), total flavonoid content (TF), total phenolic content (TP). Results were expressed as Mean ± SD (*n* = 5).

**Table 2:** Chemical compounds identified in **CE** by UHPLC–HRMS/MS.

CE–28 Identified Compounds				
Identified Compound	Chemical Formula	Exact Mass	Adduct Ion (*m*/*z*)/Monitored Negative Ion	Retention Times (Rt-Min)
**Flavonoids (Flavan-3-Ols, Flavones, Flavonols, Flavanones, Heterosides)**				
scolimoside	C_27_H_30_O_15_	594.15847	593.15121	20.23
quercetin-3-O-glucuronide	C_21_H_18_O_13_	478.07474	477.06748	20.27/24.11
kaempferol (or luteolin)-O-glucoside/isomers	C_21_H_20_O_11_	448.10056	447.09331	20.29
kaempferol-3-O-rutinoside	C_27_H_30_O_15_	594.15847	593.15122	20.33
apigenin-7-rutinoside	C_27_H_30_O_14_	578.16355	577.15630	21.03
vitexin/isovitexin	C_21_H_20_O_10_	432.10565	431.09839	21.17
apigenin-7-O-glucuronide	C_21_H_18_O_11_	446.08491	445.07763	21.21
6-methoxyluteolin	C_16_H_12_O_7_	316.05830	315.05105	22.66
apigenin	C_15_H_10_O_5_	270.05282	269.04502	23.20
kaempferol	C_15_H_10_O_6_	286.04774	285.04049	23.21
luteolin	C_15_H_10_O_6_	286.04774	285.04048	23.87
rutin (quercetin-3-O-rutinoside)	C_27_H_30_O_16_	610.15338	609.14613	24.10
hispidulin	C_16_H_12_O_6_	300.06339	299.05613	24.24
chrysoeriol	C_16_H_12_O_6_	300.06339	299.05614	25.29
chrysin	C_15_H_10_O_4_	254.05791	253.05066	25.70
**Isoflavones**				
pseudobaptigenin	C_16_H_10_O_5_	282.05282	281.04557	24.24
tectorigenin	C_16_H_12_O_6_	300.06339	299.05611	24.24
**Phenolic acids and dicarboxylic acids**				
chlorogenic acid	C_16_H_18_O_9_	354.09508	353.08783	10.44/13.80
caffeic acid	C_9_H_8_O_4_	180.04226	179.03501	14.47
azelaic acid	C_9_H_16_O_4_	188.10486	187.09761	21.24
**Depsides**				
cynarine (1,5-dicaffeoylquinic acid)	C_25_H_24_O_12_	516.12678	515.11949	20.06
**Diterpenes**				
rosmanol/epirosmanol	C_20_H_26_O_5_	346.17802	345.17077	23.80
rosmanol methyl ether	C_21_H_28_O_5_	360.19367	359.18639	26.45
rosmadial/isomers	C_20_H_24_O_5_	344.16237	343.15509	26.52
carnosol	C_20_H_26_O_4_	330.18311	329.17585	27.06
**Sesquiterpenes**				
cichorin	C_15_H_16_O_9_	340.07943	339.07218	11.94
cynaropicrin	C_19_H_22_O_6_	346.14164	345.13438	23.82
**Coumarin derivatives**				
esculetin-7-glucoside (esculin)	C_15_H_16_O_9_	340.07943	339.07218	11.94

**Table 3:** Chemical compounds identified in **RE** by UHPLC–HRMS/MS.

RE-48 Identified Compounds				
Identified Compound	Chemical Formula	Exact Mass	Adduct Ion (*m*/*z*)/Monitored Negative Ion	Retention Times (Rt-min)
**Flavonoids (Flavan-3-Ols, Flavones, Flavonols, Flavanones, Heterosides)**				
quercetin-3-O-glucuronide	C_21_H_18_O_13_	478.07474	477.06748	19.41
rutin (quercetin-3-O-rutinoside)	C_27_H_30_O_16_	610.15338	609.14613	19.51
kaempferol-3-O-rutinoside	C_27_H_30_O_15_	594.15847	593.15122	20.25
scolimoside	C_27_H_30_O_15_	594.15847	593.15121	20.25
kaempferol (or luteolin)-O-glucoside/isomers	C_21_H_20_O_11_	448.10056	447.09331	20.32/21.57/22.09
isorhamnetin-3-O-glucoside	C_22_H_22_O_12_	478.11113	477.10381	20.76
liquiritigenin/isoliquiritigenin	C_15_H_12_O_4_	256.07356	255.06631	20.94/25.03
pinostrobin	C_16_H_14_O_4_	270.08921	269.08196	20.95
apigenin-7-rutinoside	C_27_H_30_O_14_	578.16355	577.15630	21.05
apigenin-7-O-glucuronide	C_21_H_18_O_11_	446.08491	445.07763	21.22
hispidulin-7-rutinoside/isomers	C_28_H_32_O_15_	608.17412	607.16684	21.38
diosmetin-7-O-rutinoside (diosmin)	C_28_H_32_O_15_	608.17412	607.16684	21.38
hispidulin-O-glucoside/isomers	C_22_H_22_O_11_	462.11621	461.10893	21.54
naringenin	C_15_H_12_O_5_	272.06847	271.06122	22.73
hesperetin	C_16_H_14_O_6_	302.07904	301.07179	23.11
kaempferol	C_15_H_10_O_6_	286.04774	285.04049	23.22
luteolin	C_15_H_10_O_6_	286.04774	285.04048	23.89
tricin	C_17_H_14_O_7_	330.07395	329.06668	24.04
apigenin	C_15_H_10_O_5_	270.05282	269.04502	24.11
pinocembrin	C_15_H_12_O_4_	256.07356	255.06631	25.03
diosmetin	C_16_H_12_O_6_	300.06339	299.05611	25.29
2′,6-dihydroxyflavone	C_15_H_10_O_4_	254.05791	253.05066	25.75
chrysin	C_15_H_10_O_4_	254.05791	253.05066	25.76
**Isoflavones**				
formononetin	C_16_H_12_O_4_	268.07356	267.06631	20.94
medicarpin	C_16_H_14_O_4_	270.08921	269.08196	20.95
sissotrin (biochanin A 7-O-β-D-glucoside)	C_22_H_22_O_10_	446.12130	445.11404	22.04
baptigenin	C_15_H_10_O_6_	286.04774	285.04046	23.22
pratensein	C_16_H_12_O_6_	300.06339	299.05614	24.21/24.24/24.41
irisolidone	C_17_H_14_O_6_	314.07904	313.07179	24.90
biochanin A	C_16_H_12_O_5_	284.06847	283.06122	26.21
**Phenolic acids and dicarboxylic acids**				
4-hydroxy-3-methoxymandelic acid	C_9_H_10_O_5_	198.05282	197.04555	7.74
chlorogenic acid	C_16_H_18_O_9_	354.09508	353.08783	10.32/14.51
neochlorogenic acid	C_16_H_18_O_9_	354.09508	353.08783	13.82
caffeic acid	C_9_H_8_O_4_	180.04226	179.03501	14.44
ferulic acid	C_10_H_10_O_4_	194.05791	193.05066	20.31
rosmarinic acid	C_18_H_16_O_8_	360.08452	359.07726	20.93
salvianolic acid B	C_36_H_30_O_16_	718.15338	717.14610	20.93
ellagic acid	C_14_H_6_O_8_	302.00627	300.99899	21.25
azelaic acid	C_9_H_16_O_4_	188.10486	187.09761	21.26
carnosic acid	C_20_H_28_O_4_	332.19876	331.19150	29.07
**Diterpenes**				
rosmanol/epirosmanol	C_20_H_26_O_5_	346.17802	345.17077	23.83/25.55
rosmanol methyl ether	C_21_H_28_O_5_	360.19367	359.18639	26.47
rosmadial/isomers	C_20_H_24_O_5_	344.16237	343.15509	26.56/27.58
carnosic acid quinone	C_20_H_26_O_4_	329.17528	328.16803	27.05
carnosol	C_20_H_26_O_4_	330.18311	329.17585	27.05
rosmaridiphenol	C_20_H_28_O_3_	316.20384	315.19656	28.10
**Sesquiterpenes**				
cichorin	C_15_H_16_O_9_	340.07943	339.07218	11.88
**Coumarin derivatives**				
aesculetin/isomers	C_9_H_6_O_4_	178.02661	177.01935	20.93

**Table 4:** Chemical compounds identified in **TE** by UHPLC–HRMS/MS.

TE–39 Identified Compounds				
Identified Compound	Chemical Formula	Exact Mass	Adduct Ion (*m*/*z*)/ Monitored Negative ion	Retention Times (Rt-Min)
**Flavonoids (Flavan-3-Ols, Flavones, Flavonols, Flavanones, Heterosides)**				
rutin (quercetin-3-O-rutinoside)	C_27_H_30_O_16_	610.15338	609.14613	19.33
kaempferol-3-O-rutinoside	C_27_H_30_O_15_	594.15847	593.15122	20.26
scolimoside	C_27_H_30_O_15_	594.15847	593.15121	20.26
kaempferol (or luteolin)-O-glucoside/isomers	C_21_H_20_O_11_	448.10056	447.09331	20.29
cynaroside (luteolin-7-O-glucoside)	C_21_H_20_O_11_	448.10056	447.09328	20.29
quercetin-3-O-glucuronide	C_21_H_18_O_13_	478.07474	477.06748	20.52
hyperoside (quercetin-3-galactoside)	C_21_H_20_O_12_	464.09548	463.08768	20.61
vitexin (apigenin-8-C-glucoside)/isovitexin	C_21_H_20_O_10_	432.10565	431.09839	21.17
apigenin-7-O-glucuronide	C_21_H_18_O_11_	446.08491	445.07763	21.22
naringenin	C_15_H_12_O_5_	272.06847	271.06122	22.73
kaempferol	C_15_H_10_O_6_	286.04774	285.04049	23.22
luteolin	C_15_H_10_O_6_	286.04774	285.04048	23.85
apigenin/genistein	C_15_H_10_O_5_	270.05282	269.04502	24.11
tricin	C_17_H_14_O_7_	330.07395	329.06668	24.25
2′,6-dihydroxyflavone	C_15_H_10_O_4_	254.05791	253.05066	25.71
**Isoflavones**				
genistin	C_21_H_20_O_10_	432.10565	431.09837	19.62
tectorigenin	C_16_H_12_O_6_	300.06339	299.05611	24.25
pseudobaptigenin	C_16_H_10_O_5_	282.05282	281.04557	24.38
formononetin	C_16_H_12_O_4_	268.07356	267.06631	24.58
**Phenolic acids and dicarboxylic acids**				
caftaric acid	C_13_H_12_O_9_	312.04813	311.04085	9.94
chlorogenic acid	C_16_H_18_O_9_	354.09508	353.08783	10.54
neochlorogenic acid	C_16_H_18_O_9_	354.09508	353.08783	13.82
caffeic acid	C_9_H_8_O_4_	180.04226	179.03501	14.48
*p*-coumaric acid	C_9_H_8_O_3_	164.04734	163.03954	17.57
ferulic acid	C_10_H_10_O_4_	194.05791	193.05066	18.21
chicoric acid	C_22_H_18_O_12_	474.07983	473.07257	18.26
rosmarinic acid	C_18_H_16_O_8_	360.08452	359.07726	20.91
ellagic acid	C_14_H_6_O_8_	302.00627	300.99899	21.24
azelaic acid	C_9_H_16_O_4_	188.10486	187.09761	21.25
abscisic acid	C_15_H_20_O_4_	264.13616	263.12891	22.87
**Depsides**				
cynarine (1,5-dicaffeoylquinic acid)	C_25_H_24_O_12_	516.12678	515.11949	19.95/20.83
1,3-O-dicaffeoylquinic acid	C_25_H_24_O_12_	516.12678	515.11949	20.85
		**Diterpenes**		
rosmanol/epirosmanol	C_20_H_26_O_5_	346.17802	345.17077	23.80
rosmanol methyl ether	C_21_H_28_O_5_	360.19367	359.18639	26.45
rosmadial/isomers	C_20_H_24_O_5_	344.16237	343.15509	26.52
carnosol	C_20_H_26_O_4_	330.18311	329.17585	27.07
**Sesquiterpenes**				
lactucopicrin	C_23_H_22_O_7_	410.13655	409.12930	22.58
**Coumarin derivatives**				
aesculetin/isomers	C_9_H_6_O_4_	178.02661	177.01935	13.95
**Proanthocyanidins**				
procyanidin	C_30_H_26_O_13_	594.13734	593.13006	23.15

**Table 5:** Chemical compounds identified in **CHE** by UHPLC–HRMS/MS.

CHE–43 Identified Compounds				
Identified Compound	Chemical Formula	Exact Mass	Adduct Ion (*m*/*z*)/ Monitored Negative Ion	Retention Times (Rt-Min)
**Flavonoids (Flavan-3-Ols, Flavones, Flavonols, Flavanones, Heterosides)**				
catechin	C_15_H_14_O_6_	290.07904	289.07176	12.68
epicatechin	C_15_H_14_O_6_	290.07904	289.07176	16.17
chrysoeriol-7-glucoside	C_22_H_22_O_11_	462.11621	461.10893	19.34
vitexin (apigenin-8-C-glucoside)/isovitexin	C_21_H_20_O_10_	432.10565	431.09839	20.19/21.37
kaempferol-3-O-rutinoside	C_27_H_30_O_15_	594.15847	593.15122	20.25/21.58
kaempferol (or luteolin)-O-glucoside/isomers	C_21_H_20_O_11_	448.10056	447.09331	20.31
quercetin	C_15_H_10_O_7_	302.04265	301.03540	20.56/22.78
hyperoside (quercetin-3-galactoside)	C_21_H_20_O_12_	464.09548	463.08768	20.63
rutin (quercetin-3-O-rutinoside)	C_27_H_30_O_16_	610.15338	609.14613	20.65
apigetrin (apigenin-7-glucoside)	C_21_H_20_O_10_	432.10565	431.09839	21.18
apigenin-7-O-glucuronide	C_21_H_18_O_11_	446.08491	445.07763	21.22
cynaroside (luteolin-7-O-glucoside)	C_21_H_20_O_11_	448.10056	447.09328	21.51
cynarotrioside	C_33_H_40_O_20_	756.21129	755.11024	21.57
isorhamnetin-3-O-glucoside	C_22_H_22_O_12_	478.11113	477.10381	21.66
kaempferol	C_15_H_10_O_6_	286.04774	285.04049	23.23
luteolin	C_15_H_10_O_6_	286.04774	285.04048	23.23/23.86
apigenin	C_15_H_10_O_5_	270.05282	269.04502	24.12
tricin	C_17_H_14_O_7_	330.07395	329.06668	24.28
chrysoeriol	C_16_H_12_O_6_	300.06339	299.05614	24.39
chrysin	C_15_H_10_O_4_	254.05791	253.05066	25.73
2′,6-dihydroxyflavone	C_15_H_10_O_4_	254.05791	253.05066	25.78
**Isoflavones**				
genistin	C_21_H_20_O_10_	432.10565	431.09837	21.18
daidzin	C_21_H_20_O_9_	416.11073	415.10348	23.66
pratensein	C_16_H_12_O_6_	300.06339	299.05614	24.21/24.24/24.41
irisolidone	C_17_H_14_O_6_	314.07904	313.07179	24.90
biochanin A	C_16_H_12_O_5_	284.06847	283.06122	26.22
**Phenolic acids and dicarboxylic acids**				
caftaric acid	C_13_H_12_O_9_	312.04813	311.04085	9.98
chlorogenic acid	C_16_H_18_O_9_	354.09508	353.08783	10.49
neochlorogenic acid	C_16_H_18_O_9_	354.09508	353.08783	13.84
syringic acid	C_9_H_10_O_5_	198.05282	197.04555	15.83
*p*-coumaric acid	C_9_H_8_O_3_	164.04734	163.03954	17.57
chicoric acid	C_22_H_18_O_12_	474.07983	473.07257	18.27
rosmarinic acid	C_18_H_16_O_8_	360.08452	359.07726	20.92/24.56
ellagic acid	C_14_H_6_O_8_	302.00627	300.99899	21.24
azelaic acid	C_9_H_16_O_4_	188.10486	187.09761	21.28
abscisic acid	C_15_H_20_O_4_	264.13616	263.12891	21.73
**Depsides**				
cynarine (1,5-dicaffeoylquinic acid)	C_25_H_24_O_12_	516.12678	515.11949	19.97
**Diterpenes**				
rosmanol/epirosmanol	C_20_H_26_O_5_	346.17802	345.17077	23.81/25.85
rosmanol methyl ether	C_21_H_28_O_5_	360.19367	359.18639	26.45
rosmadial/isomers	C_20_H_24_O_5_	344.16237	343.15509	26.57
**Triterpenes**				
oleanolic acid	C_30_H_48_O_3_	456.36034	455.35309	31.01
**Sesquiterpenes**				
cichorin	C_15_H_16_O_9_	340.07943	339.07218	11.83
**Proanthocyanidins**				
procyanidin	C_30_H_26_O_13_	594.13734	593.13006	23.18

**Table 6:** Chemical compounds identified in **AE** by UHPLC–HRMS/MS.

AE–31 Identified Compounds				
Identified Compound	Chemical Formula	Exact Mass	Adduct Ion (*m*/*z*)/Monitored Negative Ion	Retention Times (Rt-Min)
**Flavonoids (Flavan-3-Ols, Flavones, Flavonols, Flavanones, Heterosides)**				
catechin	C_15_H_14_O_6_	290.07904	289.07176	12.68
epicatechin	C_15_H_14_O_6_	290.07904	289.07176	16.18
apigenin-7-O-glucosylglucoside	C_27_H_30_O_15_	594.15847	593.15121	17.93/21.57
hispidulin-O-glucoside/isomers	C_22_H_22_O_11_	462.11621	461.10893	19.34/21.53
apigetrin (apigenin-7-glucoside)	C_21_H_20_O_10_	432.10565	431.09839	20.21
vitexin (apigenin-8-C-glucoside)/isovitexin	C_21_H_20_O_10_	432.10565	431.09839	20.21/21.18/21.37
cynaroside (luteolin-7-O-glucoside)	C_21_H_20_O_11_	448.10056	447.09328	20.32/21.51
apigenin-7-O-glucuronide	C_21_H_18_O_11_	446.08491	445.07763	21.25
hispidulin-7-rutinoside/isomers	C_28_H_32_O_15_	608.17412	607.16684	21.39/22.33
naringenin	C_15_H_12_O_5_	272.06847	271.06122	22.71
kaempferol	C_15_H_10_O_6_	286.04774	285.04049	23.20
luteolin	C_15_H_10_O_6_	286.04774	285.04048	23.22/23.89
apigenin	C_15_H_10_O_5_	270.05282	269.04502	24.11
tricin	C_17_H_14_O_7_	330.07395	329.06668	24.27
hispidulin	C_16_H_12_O_6_	300.06339	299.05613	24.41
chrysoeriol	C_16_H_12_O_6_	300.06339	299.05614	25.29
chrysin	C_15_H_10_O_4_	254.05791	253.05066	25.72
2′,6-dihydroxyflavone	C_15_H_10_O_4_	254.05791	253.05066	25.72
**Isoflavones**				
genistin	C_21_H_20_O_10_	432.10565	431.09837	19.63
biochanin A	C_16_H_12_O_5_	284.06847	283.06122	26.22
**Phenolic acids and dicarboxylic acids**				
chlorogenic acid	C_16_H_18_O_9_	354.09508	353.08783	10.51
caffeic acid	C_9_H_8_O_4_	180.04226	179.03501	14.48
azelaic acid	C_9_H_16_O_4_	188.10486	187.09761	21.29
**Diterpenes**				
rosmanol/epirosmanol	C_20_H_26_O_5_	346.17802	345.17077	23.07/23.81
rosmanol methyl ether	C_21_H_28_O_5_	360.19367	359.18639	26.47
rosmadial/isomers	C_20_H_24_O_5_	344.16237	343.15509	26.57
carnosol	C_20_H_26_O_4_	330.18311	329.17585	27.06
rosmaridiphenol	C_20_H_28_O_3_	316.20384	315.19656	28.10
		**Triterpenes**		
oleanolic acid	C_30_H_48_O_3_	456.36034	455.35309	31.01
**Proanthocyanidins**				
procyanidin B1/B2	C_30_H_26_O_12_	578.14243	577.13514	11.38/13.97
procyanidin	C_30_H_26_O_13_	594.13734	593.13006	23.16

**Table 7:** Polyphenolic compounds quantified in vegetal extracts by UHPLC–HRMS/MS.

AP (µg/g Extract)	Vegetal Extract				
	CE	RE	TE	CHE	AE
catechin	NF	NF	5883.4	5885.7	11,854.8
epicatechin	NF	NF	878.9	895.0	6801.2
caffeic acid	3219.6	3678.2	3509.9	NF	3197.3
*p*-coumaric acid	190.4	294.3	351.6	231.3	249.6
syringic acid	72.2	150.1	149.7	65.1	NF
genistin	NF	NF	69.5	132.0	5514.9
chlorogenic acid	717.3	714.6	619.8	685.2	2032.4
ferulic acid	207.6	479.9	274.6	200.4	208.3
hyperoside	NF	NF	365.1	4250.0	15,431.3
apigenin	329.7	585.3	138.2	84.3	150.0
rutoside	191.4	105.1	446.0	1212.5	1724.7
gallic acid	NF	163.4	NF	57.4	172.3
ellagic acid	20.5	26.6	23.1	18.7	206.9
formononetin	NF	NF	632.95	61.60	NF
pinocembrin	32.7	43.1	34.0	31.9	33.2
galangin	299.2	404.4	321.5	265.7	272.5
chrysin	115.28	342.10	174.67	39.02	55.38
kaempferol	2028.4	530.9	1396.3	243.0	278.8
hesperetin	NF	20718.4	NF	NF	NF
naringin	NF	269.5	NF	NF	NF
naringenin	439.4	560.3	396.0	383.0	1375.8
quercetol	NF	NF	NF	490.98	4958.05
cinnamic acid	NF	27.40	NF	NF	NF
abscisic acid (ABA)	NF	NF	NF	NF	167.7

NF: not found.

**Table 8:** Concentrations of relevant active principles and antioxidant values.

Vegetal Extract	TP (g Tannic Acid/100 g Dry Extract)	TPA (g Chlorogenic Acid/100 g Dry Extract)	DPPH IC50 (mg/mL)	ABTS IC50 (mg/mL)	FRAP EC50 (mg/mL)
*CE*	5.7627	1.7389	0.6596	0.1588	0.5413
*RE*	31.0913	17.3293	0.0900	0.0297	0.0537
*TE*	7.0016	7.7644	0.3121	0.0752	0.2745
*CHE*	16.1272	7.7066	0.1954	0.0539	0.2012
*AE*	31.7017	24.1528	0.0537	0.0147	0.0483

**Table 9:** The comparison between antioxidant methods with ANOVA Test (ABTS/DPPH/FRAP).

	Sum of Squares	df	Mean Square	F	Sig.
**Between Groups**	0.945	2	0.473	**2.581**	0.117 *
**Within Groups**	2.198	12	0.183		
Total	3.143	14			

*. *p* > 0.05.

**Table 10:** Correlation coefficients between antioxidant methodologies.

Correlation	r	R^2^	R^2^ (%)
**ABTS vs. DPPH**	0.995	0.9900	99.0025
**ABTS vs. FRAP**	0.964	0.9293	92.9296
**DPPH vs. FRAP**	0.982	0.9643	96.4324

**DPPH**: 2,2-diphenyl-1-picryl-hydrazine; **ABTS**: 2,2′-azinobis-3-ethylbenzotiazoline-6-sulfonic acid; **FRAP**: ferric reducing antioxidant power; **r**: Pearson correlation coefficient; **R^2^**: coefficient of determination; **R^2^** (**%**): coefficient of determination expressed as a percentage.

**Table 11:** The comparison with ANOVA Test (Antioxidant Inhibition DPPH).

	Sum of Squares	df	Mean Square	F	Sig.
**Between Groups**	0.709	4	0.177	**21.885**	0.000 *
**Within Groups**	0.364	45	0.008		
Total	1.073	49			

*. *p* < 0.05.

**Table 12:** The comparison with ANOVA Test (Antioxidant Inhibition ABTS).

	Sum of Squares	df	Mean Square	F	Sig.
**Between Groups**	0.246	4	0.061	**15.960**	0.000 *
**Within Groups**	0.173	45	0.004		
Total	0.419	49			

*. *p* < 0.05.

**Table 13:** The comparison with ANOVA Test (Antioxidant FRAP).

	Sum of Squares	df	Mean Square	F	Sig.
**Between Groups**	3.804	4	0.951	**14.460**	0.000 *
**Within Groups**	2.959	45	0.066		
Total	6.763	49			

*. *p* < 0.05.

**Table 14:** Correlation coefficients between TP and antioxidant methodologies.

Correlation	r	R^2^	R^2^ (%)
**TP vs. DPPH**	−0.956	0.9139	91.3936
**TP vs. ABTS**	−0.930	0.8649	86.4900
**TP vs. FRAP**	−0.979	0.9584	95.8441

**TP**: total phenolic content; **DPPH**: 2,2-diphenyl-1-picryl-hydrazine; **ABTS**: 2,2′-azinobis-3-ethylbenzotiazoline-6-sulfonic acid; **FRAP**: ferric reducing antioxidant power; **r**: Pearson correlation coefficient; **R^2^**: coefficient of determination; **R^2^** (**%**): coefficient of determination expressed as a percentage.

**Table 15:** Correlation coefficients between TPA and antioxidant methodologies.

Correlation	r	R^2^	R^2^ (%)
**TPA vs. DPPH**	−0.973	0.9467	94.6729
**TPA vs. ABTS**	−0.980	0.9604	96.0400
**TPA vs. FRAP**	−0.965	0.9312	93.1225

**TPA**: total phenolic acids/phenolcarboxylic acids; **DPPH**: 2,2-diphenyl-1-picryl-hydrazine; **ABTS**: 2,2′-azinobis-3-ethylbenzotiazoline-6-sulfonic acid; **FRAP**: ferric reducing antioxidant power; **r**: Pearson correlation coefficient; **R^2^**: coefficient of determination; **R^2^** (**%**): coefficient of determination expressed as a percentage.

**Table 16:** Predicted binding energies and calculated ligand efficiencies for screened polyphenolic compounds on 3 target proteins relevant for potential hepatoprotective activity.

	CYP2E1		TNF-α		GPx4	
Ligand	ΔG (kcal/mol)	LE	ΔG (kcal/mol)	LE	ΔG (kcal/mol)	LE
abscisic acid	−7.807	0.4109	−7.419	0.3905	−5.499	0.2894
apigenin	−8.621	0.4311	−7.785	0.3892	−6.918	0.3459
caffeic acid	−7.644	0.5880	−6.266	0.4820	−5.491	0.4224
catechin	−8.315	0.3960	−7.951	0.3786	−5.915	0.2817
chlorogenic acid	−5.985	0.2394	−7.843	0.3137	−6.872	0.2749
chrysin	−8.800	0.4632	−7.589	0.3994	−6.641	0.3495
cinnamic acid	−8.159	0.7417	−5.980	0.5436	−5.187	0.4715
ellagic acid	−6.675	0.3034	−7.638	0.3472	−6.563	0.2983
epicatechin	−8.540	0.4067	−7.774	0.3702	−6.830	0.3252
ferulic acid	−7.253	0.5181	−6.137	0.4384	−5.137	0.3669
formononetin	−8.556	0.4278	−7.626	0.3813	−6.547	0.3273
galangin	−6.996	0.3498	−7.476	0.3738	−6.532	0.3266
gallic acid	−6.495	0.5412	−5.795	0.4829	−4.873	0.4061
genistin	−4.590	0.1481	−8.824	0.2846	−7.008	0.2261
hyperoside	−0.884	0.0268	−8.641	0.2618	−6.838	0.2072
kaempferol	−6.985	0.3326	−7.289	0.3471	−6.800	0.3238
naringenin	−8.388	0.4194	−7.228	0.3614	−6.048	0.3024
naringin	-	-	−8.640	0.2107	−7.023	0.1713
*p*-coumaric acid	−8.011	0.6676	−5.787	0.4823	−5.039	0.4199
pinocembrin	−8.942	0.4706	−7.818	0.4115	−6.738	0.3546
quercetin	−6.768	0.3076	−7.600	0.3455	−6.540	0.2973
rutin	-	-	−8.998	0.2093	−6.888	0.1602
syringic acid	−5.284	0.3774	−5.673	0.4052	−4.637	0.3312
omega-imidazolyl-dodecanoic acid	−7.776	0.4093	-	-	-	-
SPD304	-	-	−8.867	0.2217	-	-
1d4	-	-	-	-	−6.978	0.3172

**ΔG**–binding energy; **LE**–ligand efficiency.

## Data Availability

Not applicable.

## Supplementary Materials

The following supporting information can be downloaded at: https://www.mdpi.com/article/10.3390/plants11131680/s1, Figure S1: UHPLC–HRMS/MS chromatogram for CE; Figure S2: UHPLC–HRMS/MS chromatogram (a) for RE; Figure S3: UHPLC–HRMS/MS chromatogram (b) for RE; Figure S4: UHPLC–HRMS/MS chromatogram (c) for RE; Figure S5: Dendrogram for RE compounds; Figure S6: UHPLC–HRMS/MS chromatogram (a) of TE; Figure S7: UHPLC–HRMS/MS chromatogram (b) of TE; Figure S8: Dendrogram for TE compounds; Figure S9: UHPLC–HRMS/MS chromatogram (a) for CHE; Figure S10: UHPLC–HRMS/MS chromatogram (b) for CHE; Figure S11: UHPLC–HRMS/MS chromatogram (c) for CHE; Figure S12: UHPLC–HRMS/MS chromatogram (a) for AE; Figure S13: UHPLC–HRMS/MS chromatogram (b) for AE; Figure S14: Dendrogram for AE compounds; Figure S15: Boxplot Diagram–Distribution and spread of IC50 values between different methods; Figure S16: Boxplot Diagram–Distribution and spread of DPPH data set for every vegetal extract group; Figure S17: Boxplot Diagram–Distribution and spread of ABTS data set for every vegetal extract group; Figure S18: Boxplot Diagram–Distribution and spread of FRAP data set for every vegetal extract group; Figure S19: Superposition of docking poses (pink) on initial/crystal conformations (green); Figure S20: Standard curve of rutin (TF); Figure S21: Standard curve of chlorogenic acid (TPA); Figure S22: Standard curve for tannic acid (TP); Figure S23: Calibration curve for ascorbic acid (vitamin C)-Antioxidant action in 50% Ethanol; Figure S24: Calibration curve for ascorbic acid (vitamin C)-Antioxidant action in 20% Ethanol; Table S1: Validation parameters of the LC-HRMS analytical method; Table S2: Monitored compounds by full-scan-HRMS analysis and MS-MS analysis based on analytical standards.
