# Supplementary material for: The Polyphenolic Profile and Antioxidant Activity of Five Vegetal Extracts with Hepatoprotective Potential
Source: Plants (Basel). 2022 Jun 24;11(13):1680. doi: 10.3390/plants11131680 (PMC9269044; doi:10.3390/plants11131680)
Supplement: Supplementary file 1 [file plants-11-01680-s001.zip › plants-1770440-supplementary.pdf]

Article

The Polyphenolic Profile and Antioxidant Activity of Five Vegetal Extracts with Hepatoprotective Potential

Supplementary material

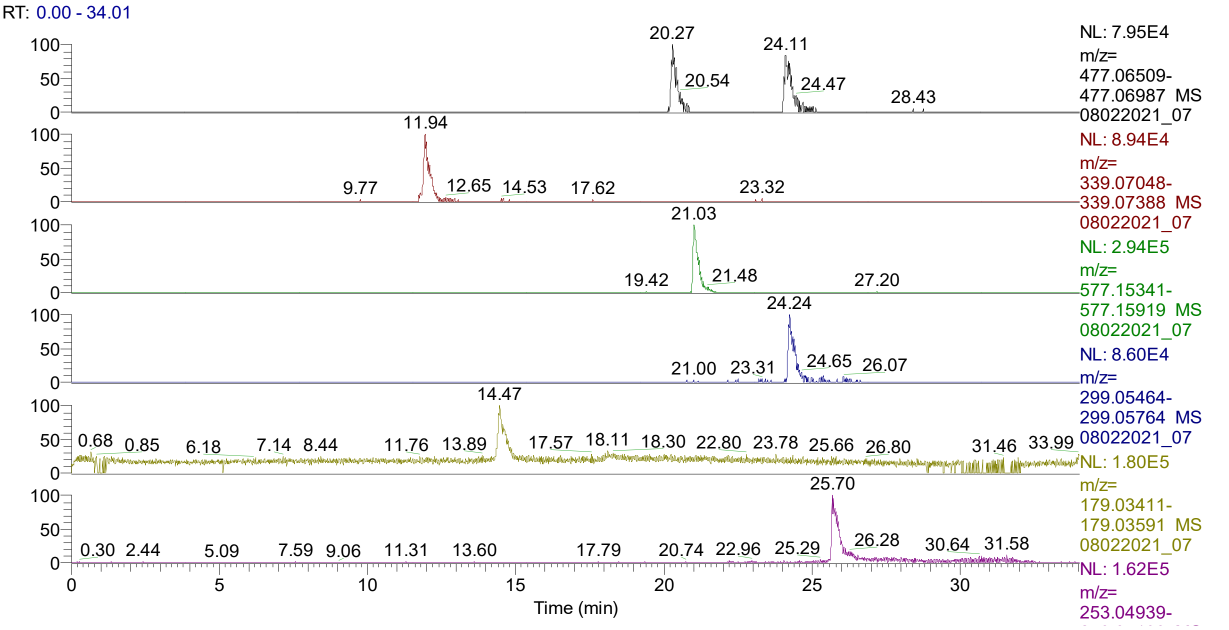

Figure S1. UHPLC-HRMS/MS chromatogram for **CE** in which were identified (top to bottom): quercetin-3-O-glucuronide (m/z: 477.06748, Rt: 20.27/24.11), esculetin-7-glucoside (m/z: 339.07218, Rt: 11.94), apigenin-7-rutinoside (m/z: 577.15630, Rt: 21.03), pratensein (m/z: 299.05614, Rt: 24.24), caffeic acid (m/z: 179.03501, Rt: 14.47), chrysin (m/z: 253.05066, Rt: 25.70).

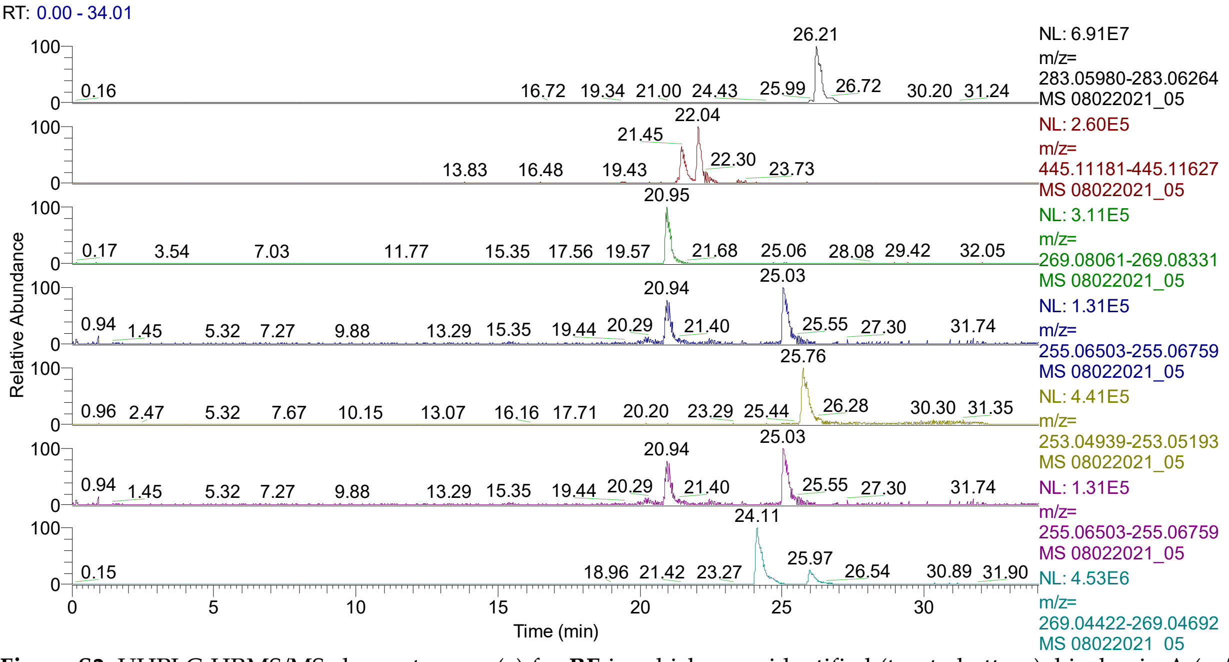

Figure S2. UHPLC-HRMS/MS chromatogram (a) for **RE** in which were identified (top to bottom): biochanin A (m/z: 283.06122, Rt: 26.21), sissotrin/biochanin A-7-O-β-D-glucoside (m/z: 445.11404, Rt: 22.04), medicarpin (m/z: 269.08196, Rt: 20.95), pinocembrin (m/z: 255.06631, Rt: 25.03), chrysin (m/z: 253.05066, Rt: 25.76), liquiritigenin/isoliquiritigenin (m/z: 255.06631, Rt: 20.94/25.03), galangin (m/z: 269.04930, Rt: 24.11).

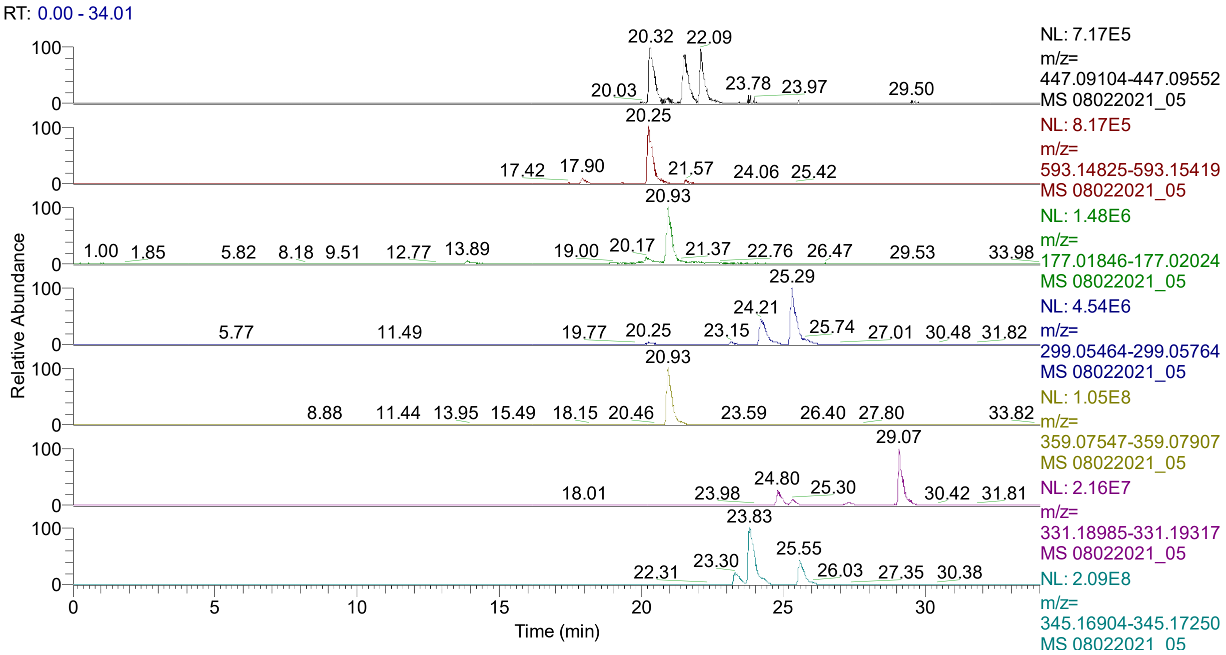

Figure S3. UHPLC-HRMS/MS chromatogram (b) for **RE** in which were identified (top to bottom): kaempferol (or luteolin)-O-glucoside/isomers (m/z: 447.09331, Rt: 20.32/22.09/21.57), scolimoside (m/z: 593.15121, Rt: 20.25), aesculetin (m/z: 177.01935, Rt: 20.93), chrysoeriol (m/z: 299.05614, Rt: 25.29) pratensein (m/z: 299.05614, Rt: 24.21), rosmarinic acid (m/z: 359.07726, Rt: 20.93), carnosic acid (m/z: 331.19150, Rt: 29.07), rosmanol (m/z: 345.17077, Rt: 23.83/25.55).

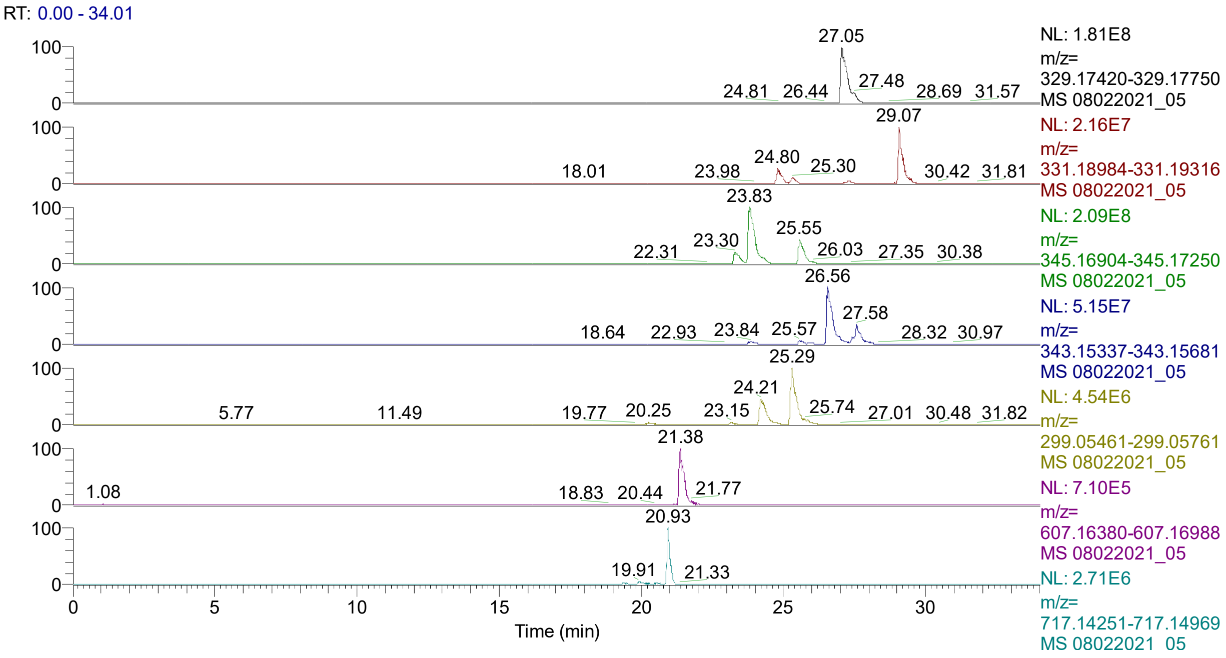

Figure S4. UHPLC-HRMS/MS chromatogram (c) for **RE** in which were identified (top to bottom): carnosol (m/z: 329.17585, Rt: 27.05), carnosic acid (m/z: 331.19150, Rt: 29.07), rosmanol (m/z: 345.17077, Rt: 23.83/25.55), rosmadial (m/z: 343.15509, Rt: 26.56/27.58), diosmetin (m/z: 299.05611, Rt: 25.29), diosmetin-7-O-rutinoside/diosmin (m/z: 607.16684, Rt: 21.38), salvianolic acid B (m/z: 717.14610, Rt: 20.93).

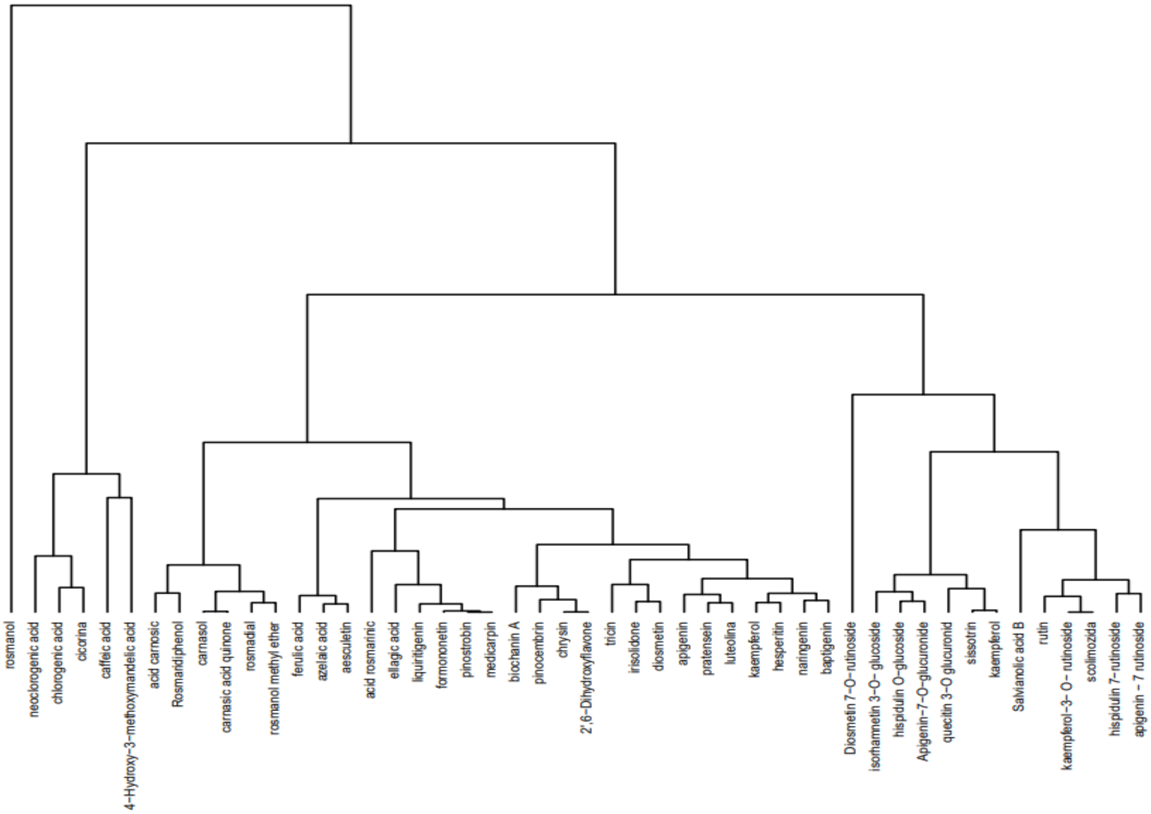

Figure S5. Dendrogram for **RE** compounds representative for the profile of polyphenolic derivatives obtained by the hierarchical grouping method Ward, HCA (Hierarchical Cluster Analysis).

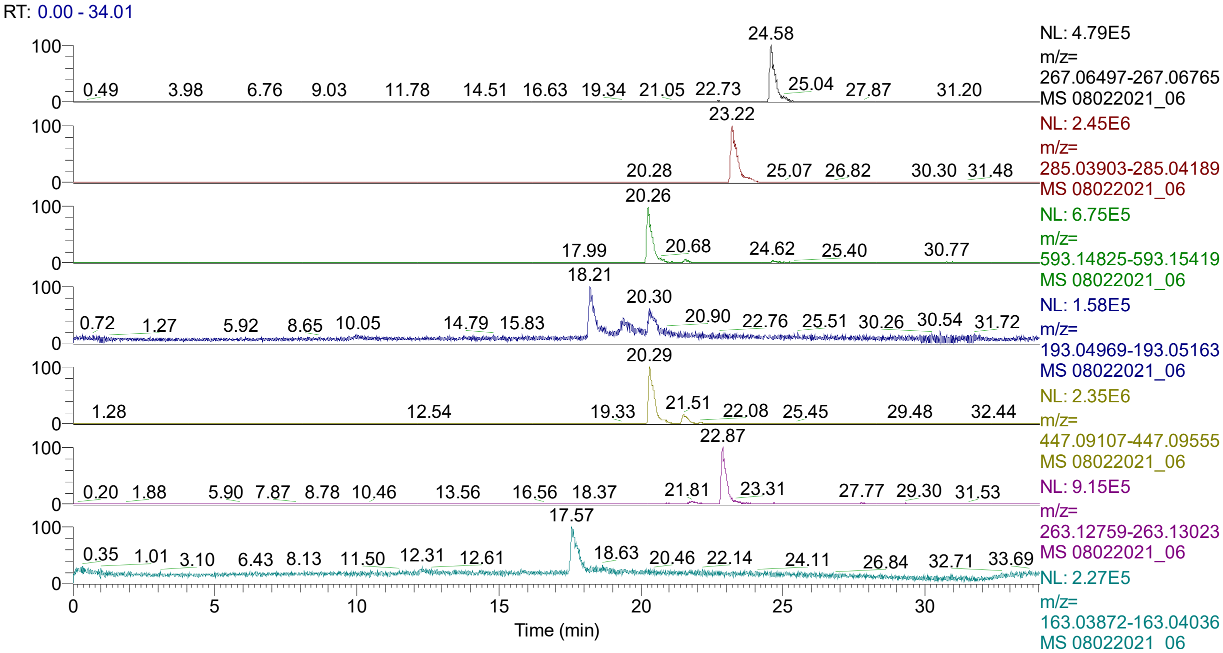

Figure S6. UHPLC-HRMS/MS chromatogram (a) of **TE** in which were identified (top to bottom): formononetin (m/z: 267.06631, Rt: 24.58), baptigenin (m/z: 285.04046, Rt: 23.22), kaempferol-3-O-rutinoside (m/z: 593.15122, Rt: 20.26), ferulic acid (m/z: 193.05066, Rt: 18.21), kaempferol (or luteolin)-O-glucoside (m/z: 447.09331, Rt: 20.29), abscisic acid (m/z: 263.12891, Rt: 22.87), p-coumaric acid (m/z: 163.03954, Rt: 17.57).

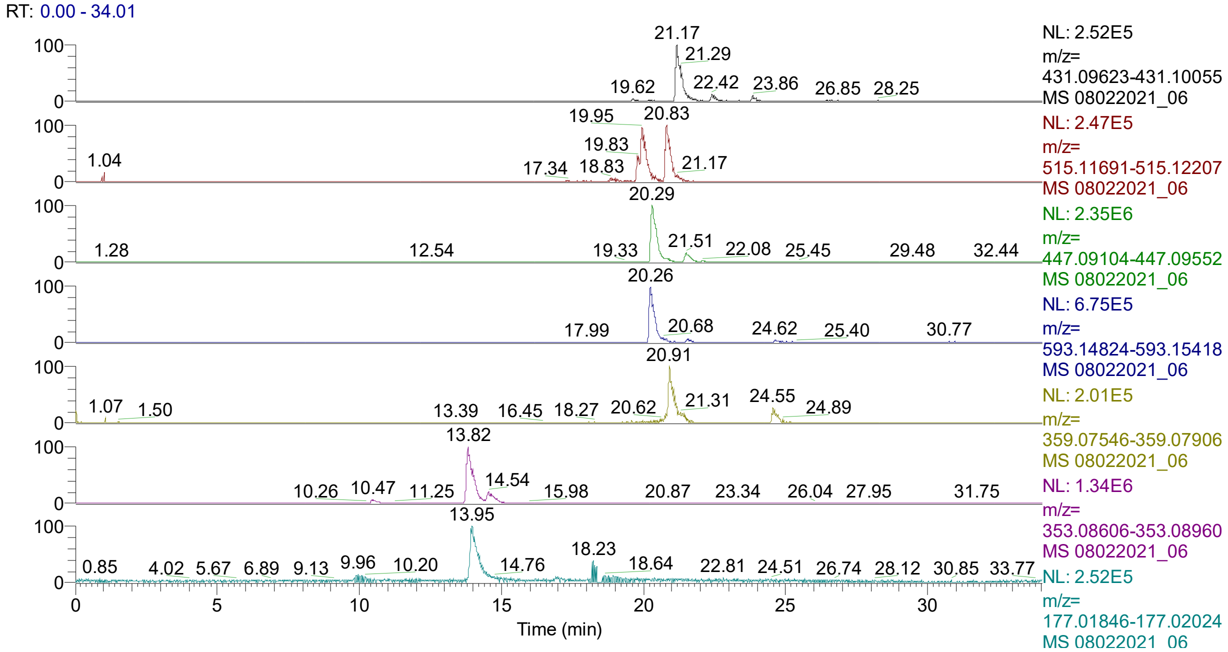

Figure S7. UHPLC-HRMS/MS chromatogram (b) of **TE** in which were identified (top to bottom): vitexin (m/z: 431.09839, Rt: 21.17), cynarine/1,5-dicaffeoylquinic acid (m/z: 515.11949, Rt: 19.95/20.83), cynaroside/luteolin-7-O-glucoside (m/z: 447.09328, Rt: 20.29), scolimoside (m/z: 593.15121, Rt: 20.26), rosmarinic acid (m/z: 359.07726, Rt: 20.91), neochlorogenic acid (m/z: 353.08783, Rt: 13.82), aesculetin (m/z: 177.01935, Rt: 13.95).

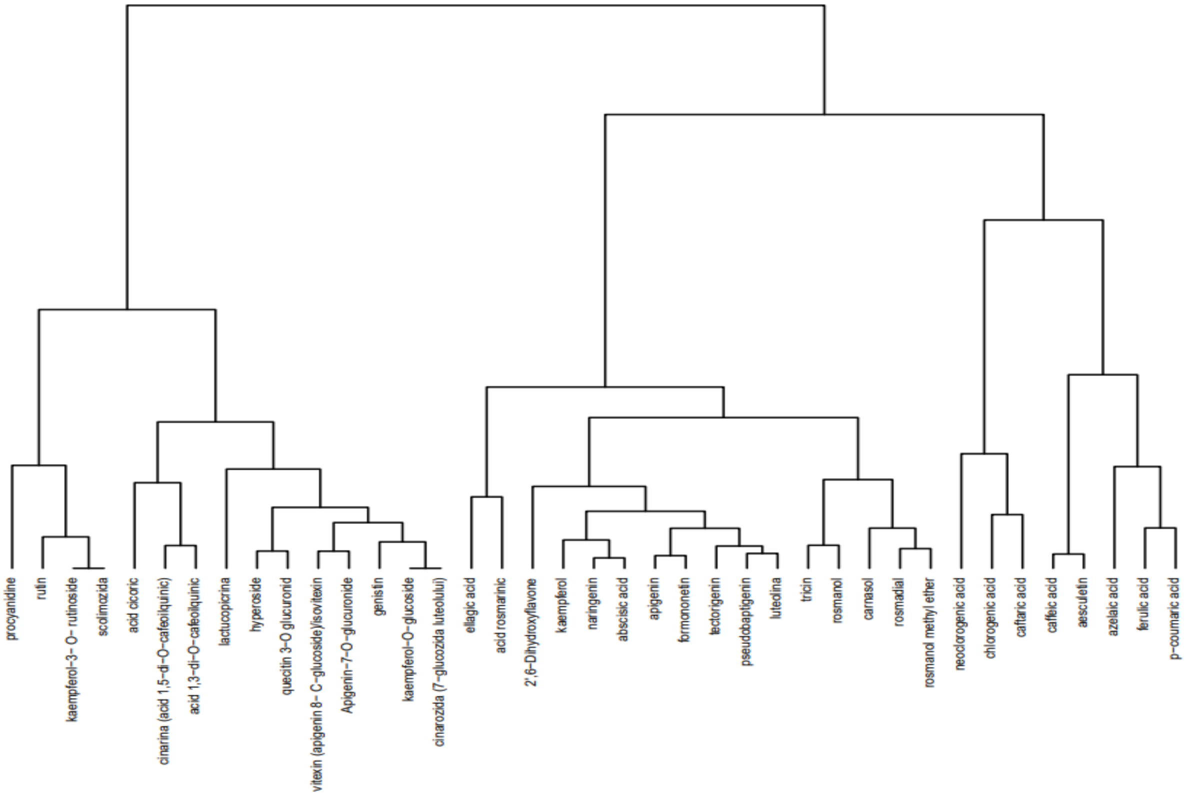

Figure S8. Dendrogram for **TE** compounds representative for the profile of polyphenolic derivatives obtained by the hierarchical grouping method Ward, HCA (Hierarchical Cluster Analysis).

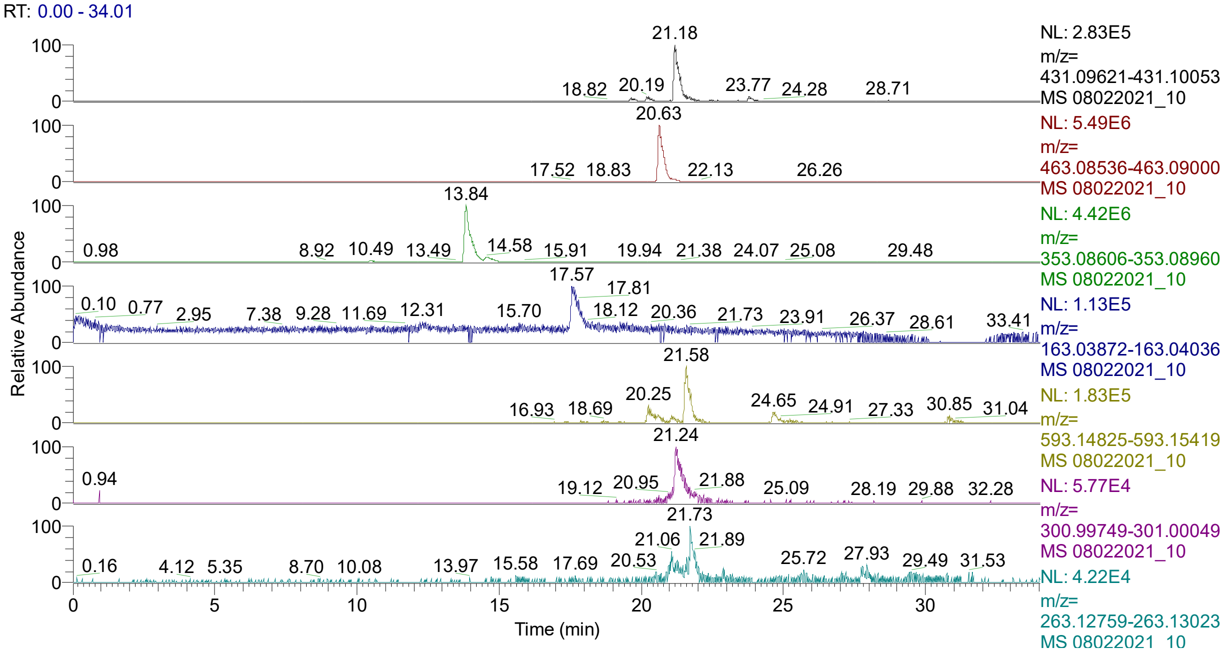

Figure S9. UHPLC-HRMS/MS chromatogram (a) for **CHE** in which were identified (top to bottom): genistin (m/z: 431.09837, Rt: 21.18), hyperoside/quercetin-3-galactoside (m/z: 463.08768, Rt: 20.63,), neochlorogenic acid (m/z: 353.08783, Rt: 13.84), p-coumaric acid (m/z: 163.03954, Rt: 17.57), kaempferol-3-O-rutinoside (m/z: 593.15122, Rt: 20.25/21.58), ellagic acid (m/z: 300.99899, Rt: 21.24), abscisic acid (m/z: 263.12891, Rt: 21.73).

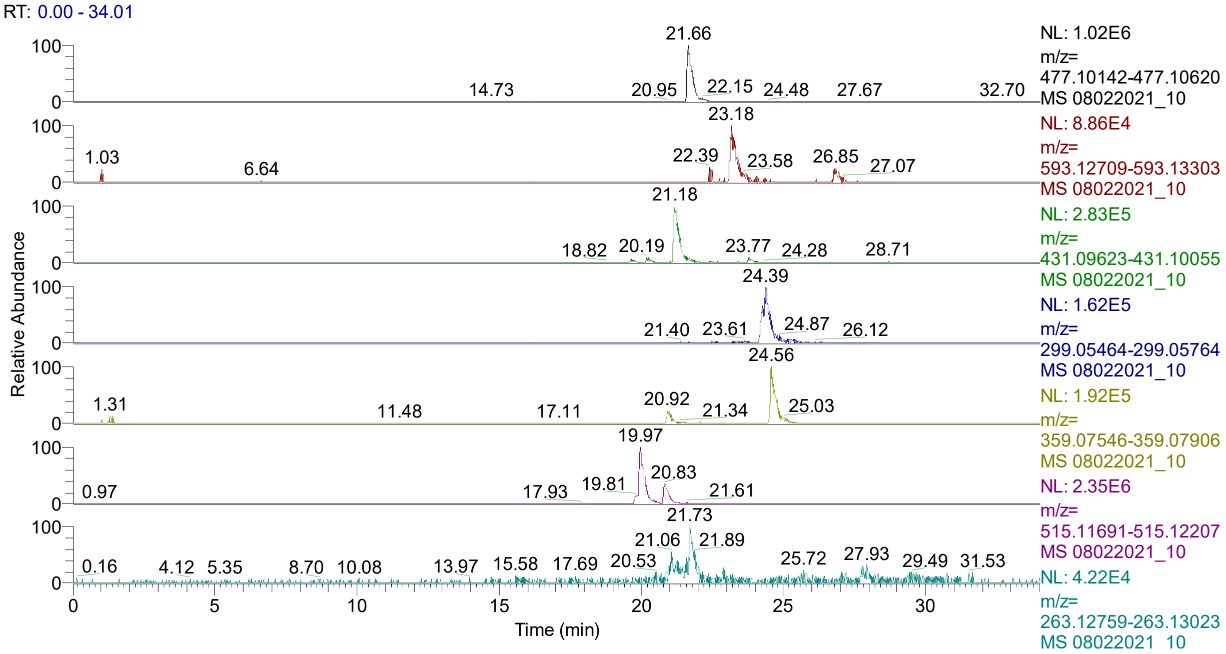

Figure S10. UHPLC-HRMS/MS chromatogram (b) for **CHE** in which were identified (top to bottom): isorhamnetin-3-O-glucoside (m/z: 477.10381, Rt: 21.66), procyanidin (m/z: 593.13006, Rt: 23.18), apigetrin /apigenin-7-glucoside (m/z: 431.09839, Rt: 21.18), chrysoeriol (m/z: 299.05614, Rt: 24.39), rosmarinic acid + isomer (m/z: 359.07726, Rt: 20.92/24.56), 1,5- and 1,3-dicaffeoylquinic acid (m/z: 515.11949, Rt: 19.97), abscisic acid (m/z: 263.12891, Rt: 21.73).

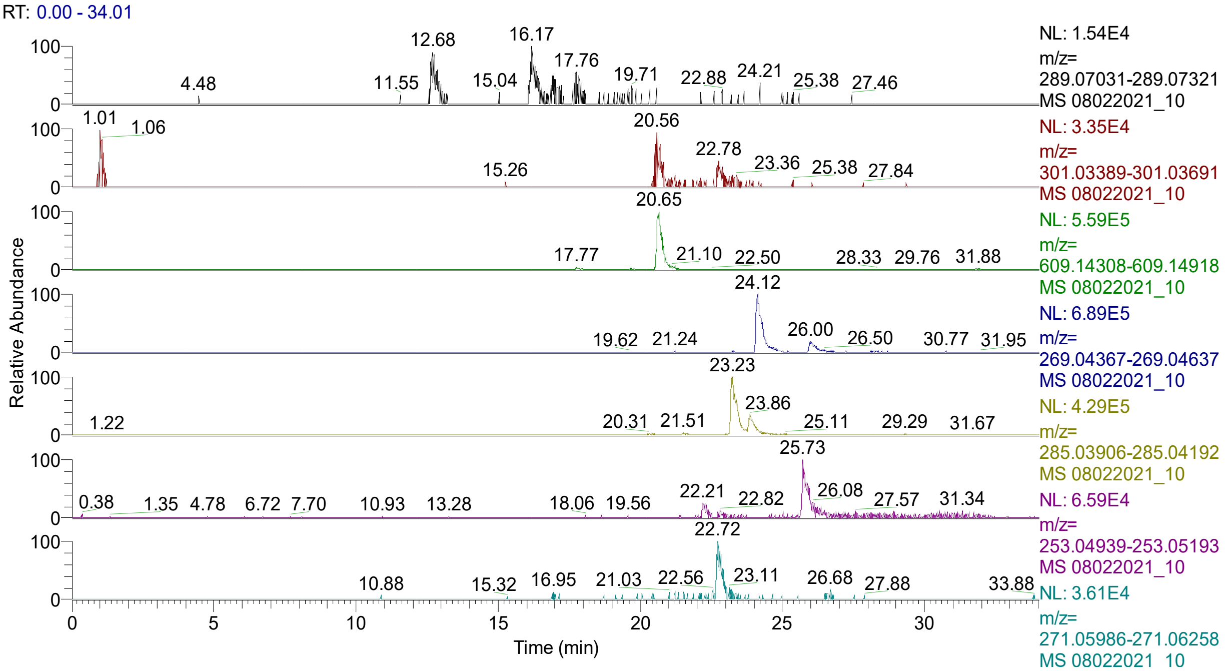

Figure S11. UHPLC-HRMS/MS chromatogram (c) for **CHE** in which were identified (top to bottom): catechin/epicatechin (m/z: 289.07176, Rt: 12.68, Rt: 16.17), quercetin (m/z: 301.03540, Rt: 20.56/22.78), rutin (m/z: 609.14613, Rt: 20.65), apigenin (m/z: 269.04502, Rt: 24.12), kaempferol (m/z: 285.04049, Rt: 23.23), chrysin (m/z: 253.05066, Rt: 25.73), naringenin (m/z: 271.06122, Rt: 22.72).

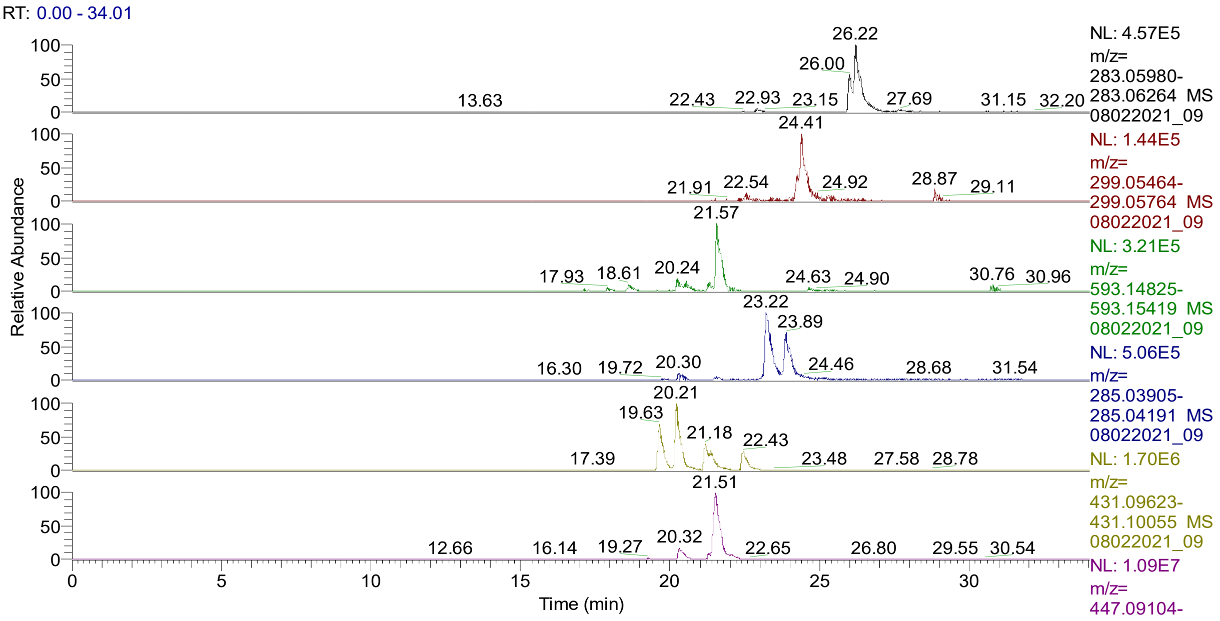

Figure S12. UHPLC-HRMS/MS chromatogram (a) for **AE** in which were identified (top to bottom): biochanin A (m/z: 283.06122, Rt: 26.22), pratensein (m/z: 299.05614, Rt: 24.41), apigenin-7-O-glucosylglucoside (m/z: 593.15121, Rt: 17.93/21.57), luteolin (m/z: 285.04048, Rt: 23.22/23.89), vitexin/isovitexin (m/z: 431.09839, Rt: 20.21), luteolin-O-glucoside/isomers (m/z: 447.09331, Rt: 20.32/21.51).

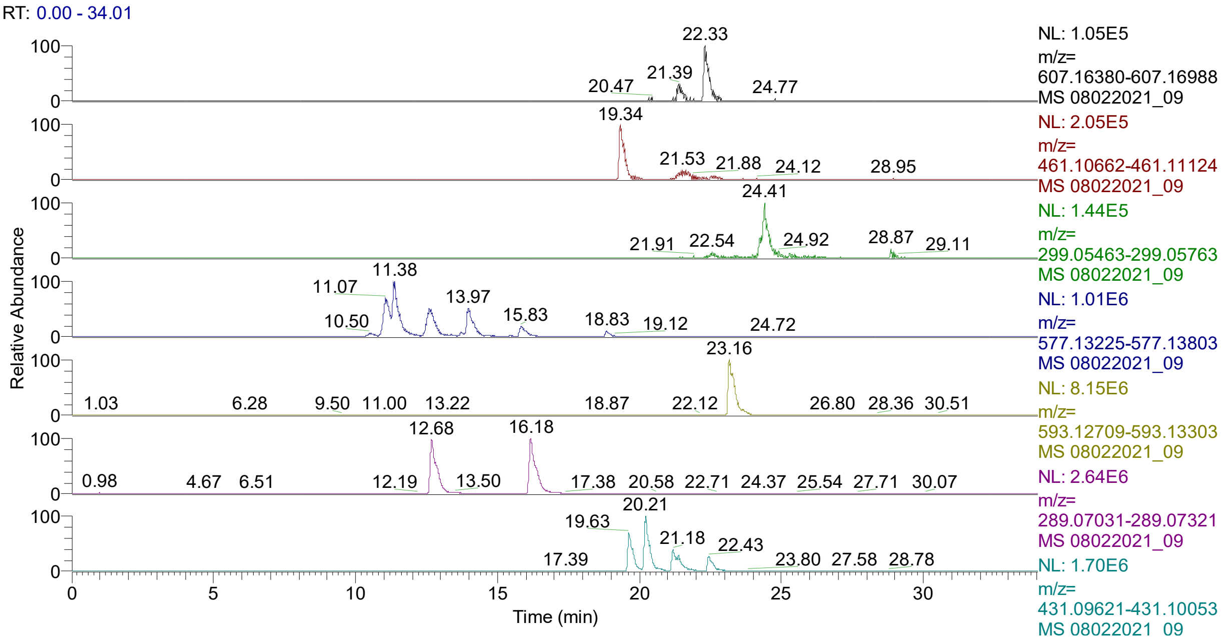

Figure S13. UHPLC-HRMS/MS chromatogram (b) for **AE** in which were identified (top to bottom): hispidulin-7-rutinoside/isomers (m/z: 607.16684, Rt: 21.39/22.33), hispidulin-O-glucoside/isomers (m/z: 461.10893, Rt: 19.34/21.53), hispidulin (m/z: 299.05613, Rt: 24.41), procyanidin B1/B2 (m/z: 577.13514, Rt: 11.38/13.97), procyanidin (m/z: 593.13006, Rt: 23.16), catechin/epicatechin (m/z: 289.07176, Rt: 12.68, Rt: 16.18), genistin/apigenin-7-glucoside/apigenin-8-C-glucoside/isomers (m/z: 431.09830, Rt: 19.63, Rt: 20.21, Rt: 21.18).

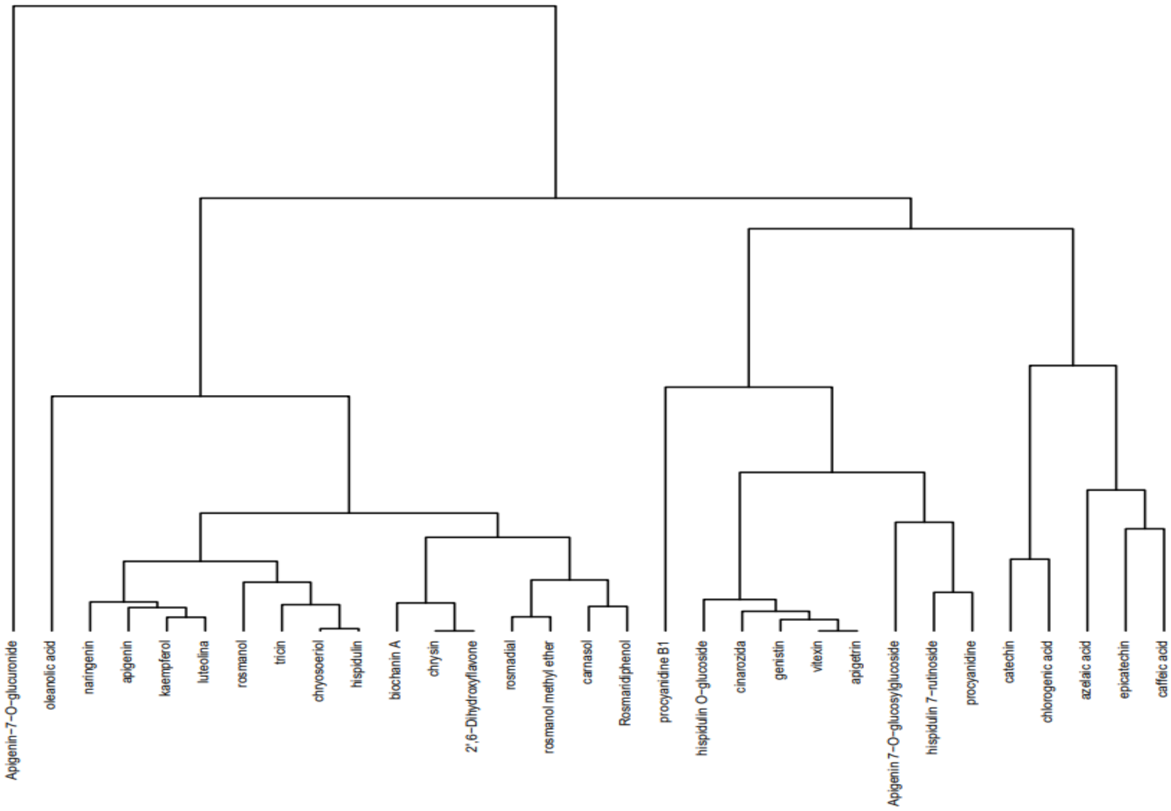

Figure S14. Dendrogram for **AE** compounds representative for the profile of polyphenolic derivatives obtained by the hierarchical grouping method Ward, HCA (Hierarchical Cluster Analysis).

Table S1. Antioxidant activity expressed as IC50 value.

Vegetal extract	DPPH method (mg/mL)	ABTS method (mg/mL)	FRAP method (mg/mL)
CE	0.6596	0.1588	0.5413
RE	0.0900	0.0297	0.0537
TE	0.3121	0.0752	0.2745
CHE	0.1954	0.0539	0.2012
AE	0.0537	0.0147	0.0483

$p>0.05$ (significance level: 0.05).

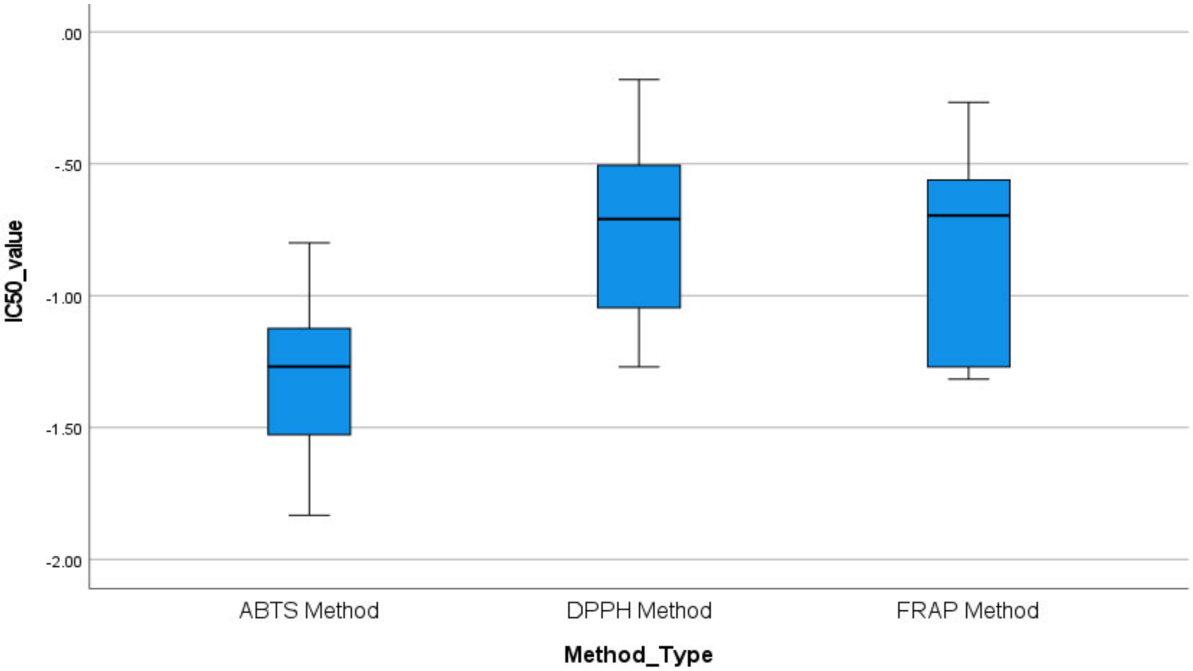

Figure S15. Boxplot Diagram – Distribution and spread of IC50 values between different methods. **(*assuming the normality of the data and the absence of outliers).**

Table S2. Pearson's correlation coefficient between different antioxidant methodologies.

		ABTS	DPPH	FRAP
**ABTS**	**Pearson Correlation**	1	.995*	.964*
	Sig. (2-tailed)		0.000	0.008
	N	5	5	5
**DPPH**	**Pearson Correlation**	.995*	1	.982*
	Sig. (2-tailed)	0.000		0.003
	N	5	5	5
**FRAP**	**Pearson Correlation**	.964*	.982*	1
	Sig. (2-tailed)	0.008	0.003	
	N	5	5	5

*. Correlation is significant at the 0.05 level (2-tailed).

Table S3. Multiple comparisons between groups for DPPH method (Games-Howell Post-Hoc Test - unequal variances).

(I) Vegetal extract group	(J) Vegetal extract group	Mean Difference (I-J)	Std. Error	Sig.	95% Confidence Interval	
					Lower Bound	Upper Bound
DPPH *Cynara*	DPPH *Rosmarinus*	-.1900500*	0.0371136	0.004	-0.313823	-0.066277
	DPPH *Agrimonia*	-.3312400*	0.0481896	0.000	-0.492484	-0.169996
	DPPH *Taraxacum*	-.0386900*	0.0120439	0.045	-0.076658	-0.000722
	DPPH *Cichorium*	-.0910200*	0.0168290	0.002	-0.145561	-0.036479
DPPH *Rosmarinus*	DPPH *Cynara*	.1900500*	0.0371136	0.004	0.066277	0.313823
	DPPH *Agrimonia*	-0.1411900	0.0603811	0.181	-0.325059	0.042679
	DPPH *Taraxacum*	.1513600*	0.0383236	0.017	0.026522	0.276198
	DPPH *Cichorium*	0.0990300	0.0400857	0.161	-0.028249	0.226309
DPPH *Agrimonia*	DPPH *Cynara*	.3312400*	0.0481896	0.000	0.169996	0.492484
	DPPH *Rosmarinus*	0.1411900	0.0603811	0.181	-0.042679	0.325059
	DPPH *Taraxacum*	.2925500*	0.0491276	0.001	0.130629	0.454471
	DPPH *Cichorium*	.2402200*	0.0505141	0.004	0.076818	0.403622
DPPH *Taraxacum*	DPPH *Cynara*	.0386900*	0.0120439	0.045	0.000722	0.076658
	DPPH *Rosmarinus*	-.1513600*	0.0383236	0.017	-0.276198	-0.026522
	DPPH *Agrimonia*	-.2925500*	0.0491276	0.001	-0.454471	-0.130629
	DPPH *Cichorium*	-0.0523300	0.0193519	0.098	-0.111687	0.007027
DPPH *Cichorium*	DPPH *Cynara*	.0910200*	0.0168290	0.002	0.036479	0.145561
	DPPH *Rosmarinus*	-0.0990300	0.0400857	0.161	-0.226309	0.028249
	DPPH *Agrimonia*	-.2402200*	0.0505141	0.004	-0.403622	-0.076818
	DPPH *Taraxacum*	0.0523300	0.0193519	0.098	-0.007027	0.111687

*. The mean difference is significant at the 0.05 level.

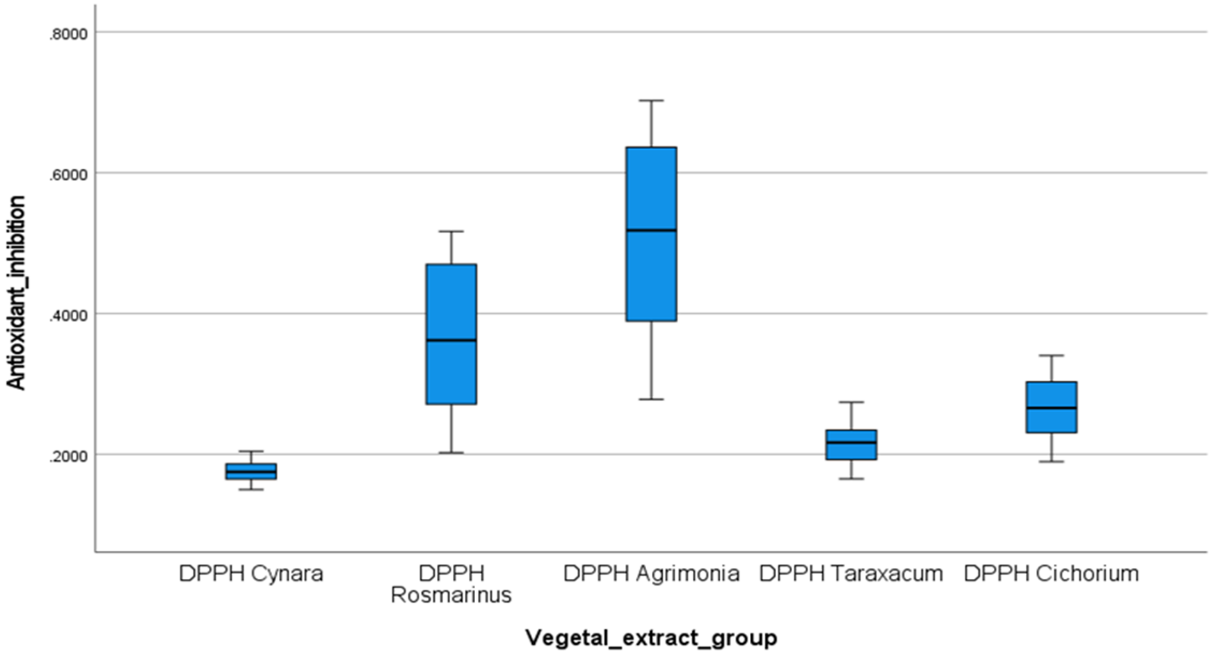

Figure S16. Boxplot Diagram – Distribution and spread of DPPH data set for every vegetal extract group. **(*mandatory assumptions were tested in order to apply statistical analysis).**

Table S41. Multiple comparisons between groups for ABTS method (Games-Howell Post-Hoc Test - unequal variances).

(I) Vegetal extract group	(J) Vegetal extract group	Mean Difference (I-J)	Std. Error	Sig.	95% Confidence Interval	
					Lower Bound	Upper Bound
ABTS *Cynara*	ABTS *Rosmarinus*	-0.0169300	0.0263381	0.966	-0.096574	0.062714
	ABTS *Agrimonia*	-.1376100*	0.0373452	0.017	-0.253682	-0.021538
	ABTS *Taraxacum*	.0689800*	0.0201256	0.032	0.005072	0.132888
	ABTS *Cichorium*	0.0334400	0.0212481	0.535	-0.032579	0.099459
ABTS *Rosmarinus*	ABTS *Cynara*	0.0169300	0.0263381	0.966	-0.062714	0.096574
	ABTS *Agrimonia*	-.1206800*	0.0375135	0.041	-0.237107	-0.004253
	ABTS *Taraxacum*	.0859100*	0.0204362	0.009	0.020925	0.150895
	ABTS *Cichorium*	0.0503700	0.0215424	0.190	-0.016661	0.117401
ABTS *Agrimonia*	ABTS *Cynara*	.1376100*	0.0373452	0.017	0.021538	0.253682
	ABTS *Rosmarinus*	.1206800*	0.0375135	0.041	0.004253	0.237107
	ABTS *Taraxacum*	.2065900*	0.0334456	0.001	0.096722	0.316458
	ABTS *Cichorium*	.1710500*	0.0341328	0.003	0.060439	0.281661
ABTS *Taraxacum*	ABTS *Cynara*	-.0689800*	0.0201256	0.032	-0.132888	-0.005072
	ABTS *Rosmarinus*	-.0859100*	0.0204362	0.009	-0.150895	-0.020925
	ABTS *Agrimonia*	-.2065900*	0.0334456	0.001	-0.316458	-0.096722
	ABTS *Cichorium*	-0.0355400	0.0132448	0.099	-0.075884	0.004804
ABTS *Cichorium*	ABTS *Cynara*	-0.0334400	0.0212481	0.535	-0.099459	0.032579
	ABTS *Rosmarinus*	-0.0503700	0.0215424	0.190	-0.117401	0.016661
	ABTS *Agrimonia*	-.1710500*	0.0341328	0.003	-0.281661	-0.060439
	ABTS *Taraxacum*	0.0355400	0.0132448	0.099	-0.004804	0.075884

*. The mean difference is significant at the 0.05 level.

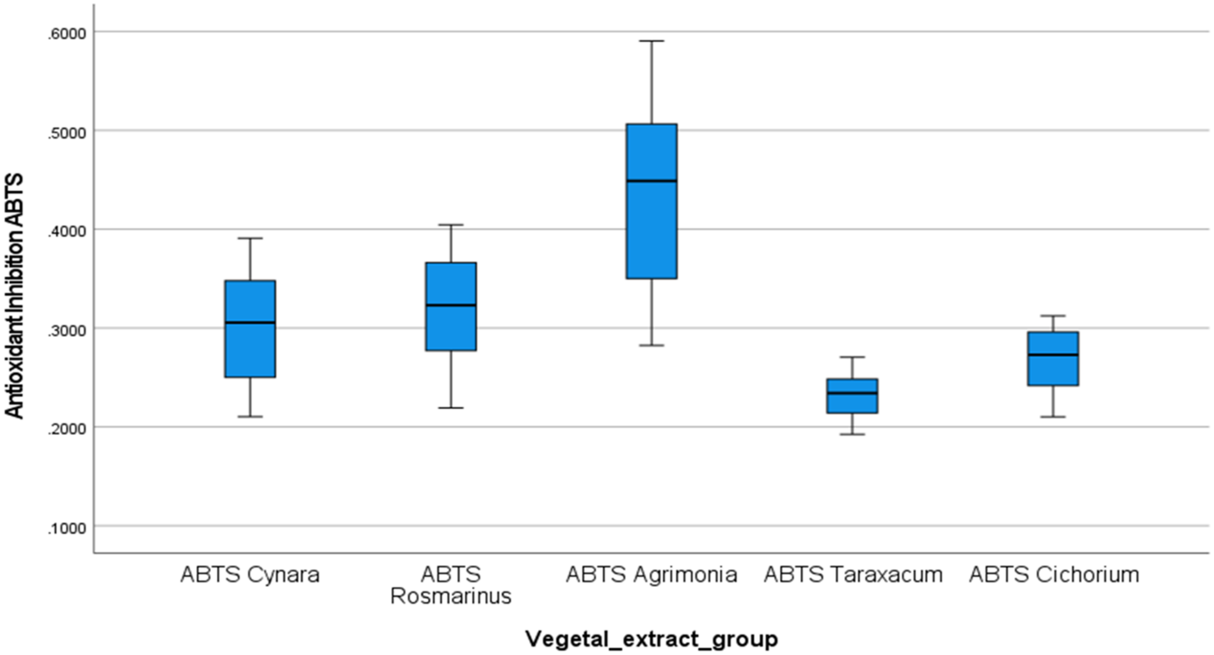

Figure S17. Boxplot Diagram – Distribution and spread of ABTS data set for every vegetal extract group **(*were applied tests of normality, homogeneity of variances, linearity tests and absence of outliers).**

Table S5. Multiple comparisons between groups for FRAP method (Tukey HSD Post-Hoc Test - equal variances).

(I) Vegetal extract group	(J) Vegetal extract group	Mean Difference (I-J)	Std. Error	Sig.	95% Confidence Interval	
					Lower Bound	Upper Bound
FRAP *Cynara*	FRAP *Rosmarinus*	-.70163*	0.11469	0.000	-1.0275	-0.3758
	FRAP *Agrimonia*	-.70736*	0.11469	0.000	-1.0332	-0.3815
	FRAP *Taraxacum*	-0.22463	0.11469	0.303	-0.5505	0.1012
	FRAP *Cichorium*	-.33381*	0.11469	0.042	-0.6597	-0.0079
FRAP *Rosmarinus*	FRAP *Cynara*	.70163*	0.11469	0.000	0.3758	1.0275
	FRAP *Agrimonia*	-0.00573	0.11469	1.000	-0.3316	0.3201
	FRAP *Taraxacum*	.47700*	0.11469	0.001	0.1511	0.8029
	FRAP *Cichorium*	.36782*	0.11469	0.020	0.0419	0.6937
FRAP *Agrimonia*	FRAP *Cynara*	.70736*	0.11469	0.000	0.3815	1.0332
	FRAP *Rosmarinus*	0.00573	0.11469	1.000	-0.3201	0.3316
	FRAP *Taraxacum*	.48273*	0.11469	0.001	0.1569	0.8086
	FRAP *Cichorium*	.37355*	0.11469	0.017	0.0477	0.6994
FRAP *Taraxacum*	FRAP *Cynara*	0.22463	0.11469	0.303	-0.1012	0.5505
	FRAP *Rosmarinus*	-.47700*	0.11469	0.001	-0.8029	-0.1511
	FRAP *Agrimonia*	-.48273*	0.11469	0.001	-0.8086	-0.1569
	FRAP *Cichorium*	-0.10918	0.11469	0.875	-0.4351	0.2167
FRAP *Cichorium*	FRAP *Cynara*	.33381*	0.11469	0.042	0.0079	0.6597
	FRAP *Rosmarinus*	-.36782*	0.11469	0.020	-0.6937	-0.0419
	FRAP *Agrimonia*	-.37355*	0.11469	0.017	-0.6994	-0.0477
	FRAP *Taraxacum*	0.10918	0.11469	0.875	-0.2167	0.4351

*. The mean difference is significant at the 0.05 level.

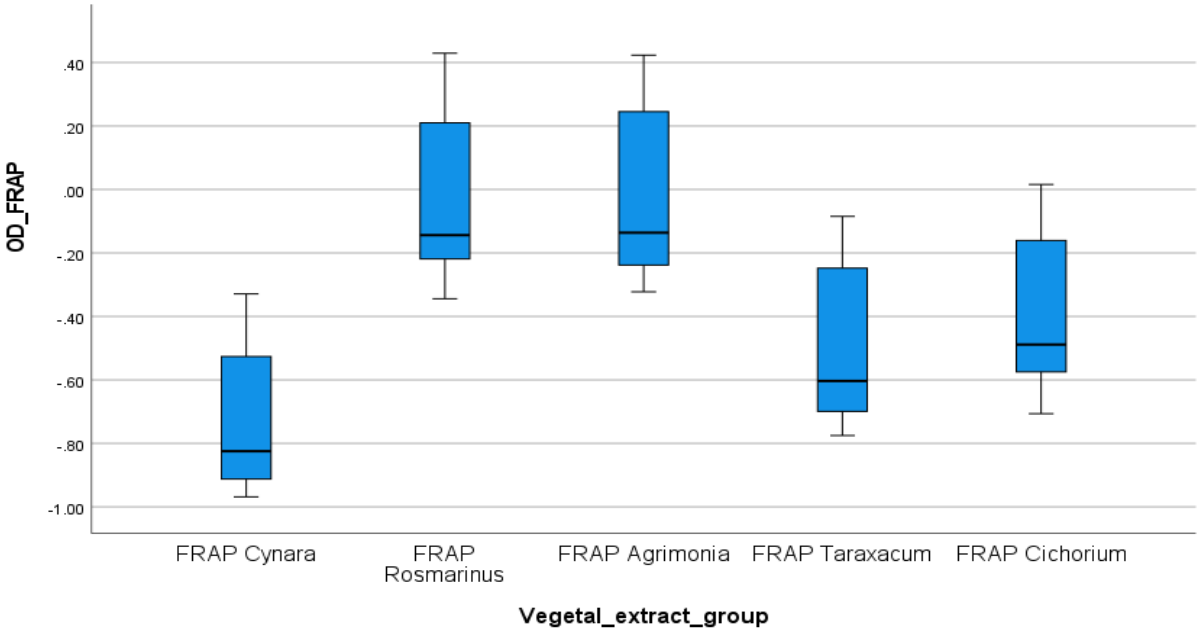

Figure S18. Boxplot Diagram – Distribution and spread of FRAP data set for every vegetal extract group.
(*normally distributed data set).

Table S6. Pearson's correlation coefficient between total phenolic compounds (TP) and total antioxidant activity.

		TP_value	ABTS IC50	DPPH IC50	FRAP EC50
**TP value**	**Pearson Correlation**	1	-.930*	-.956*	-.979*
	Sig. (2-tailed)		0.022	0.011	0.004
	N	5	5	5	5

*. Correlation is significant at the 0.05 level (2-tailed).

Table S7. Pearson's correlation coefficient between total phenolic acids (TPA) and total antioxidant activity.

		TPA_value	ABTS IC50	DPPH IC50	FRAP EC50
**TPA value**	**Pearson Correlation**	1	-.980*	-.973*	-.965*
	Sig. (2-tailed)		0.003	0.005	0.008
	N	5	5	5	5

*. Correlation is significant at the 0.05 level (2-tailed).

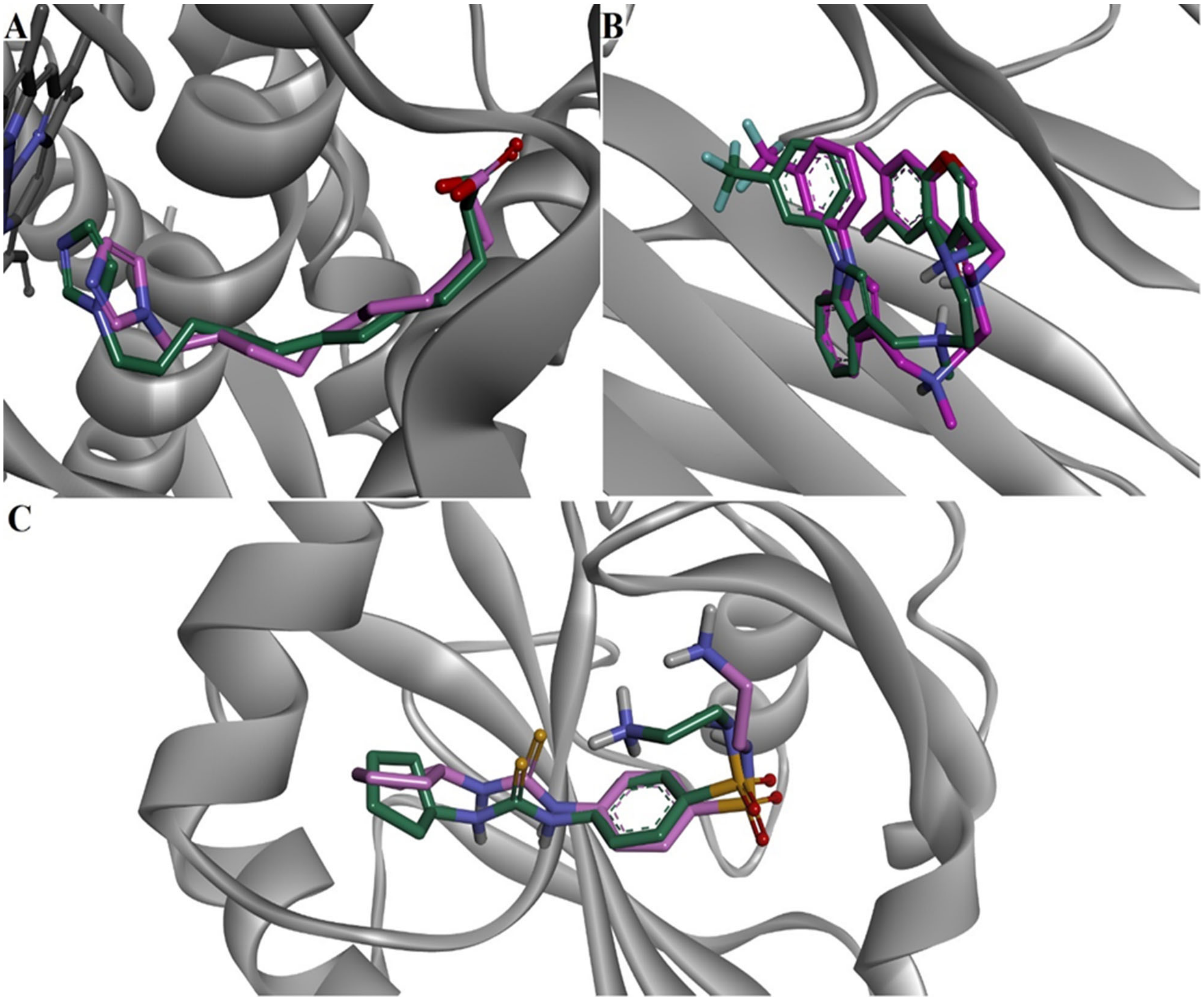

Figure S19. Superposition of docking poses (pink) on initial/crystal conformations (green). **A** – CYP2E1 inhibitor omega-imidazolyl-dodecanoic acid; **B** – TNF-α inhibitor SPD304; **C** – GPx4 allosteric activator 1d4.

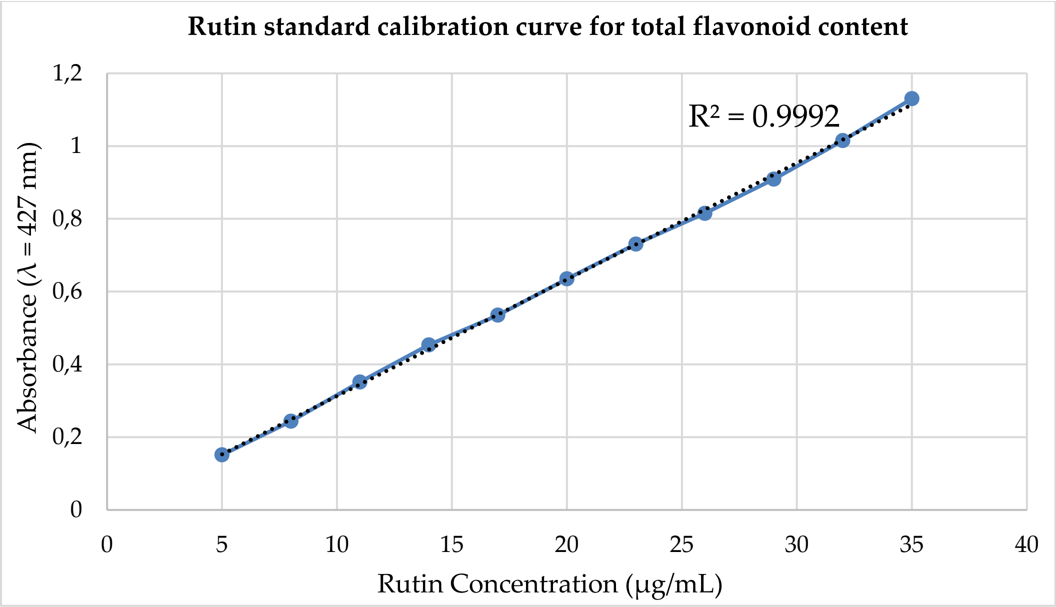

Figure S20. Standard curve of rutin (TF).

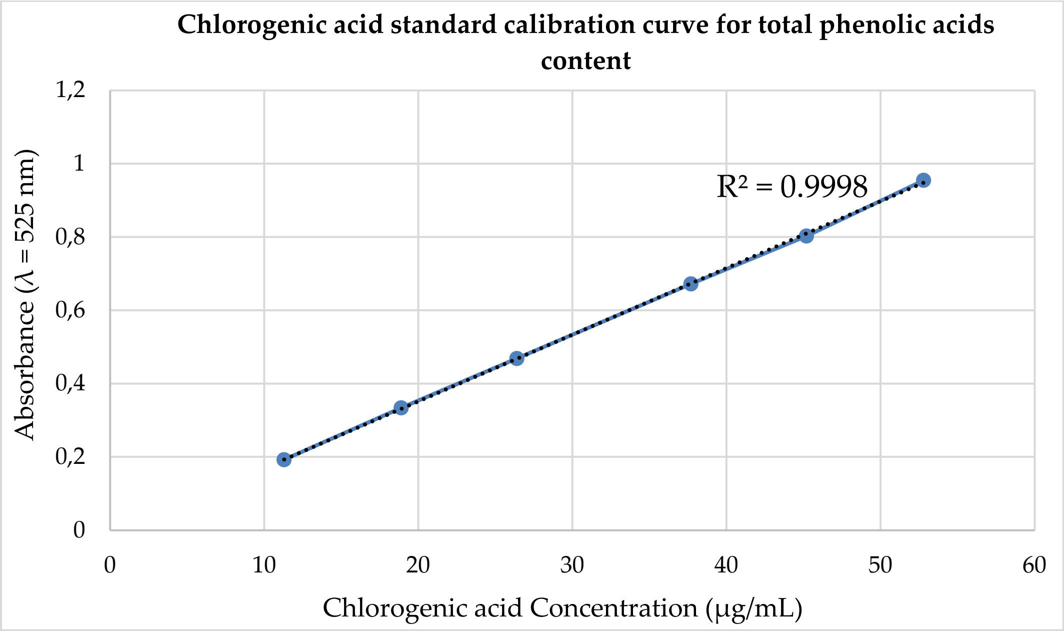

Figure S21. Standard curve of chlorogenic acid (TPA).

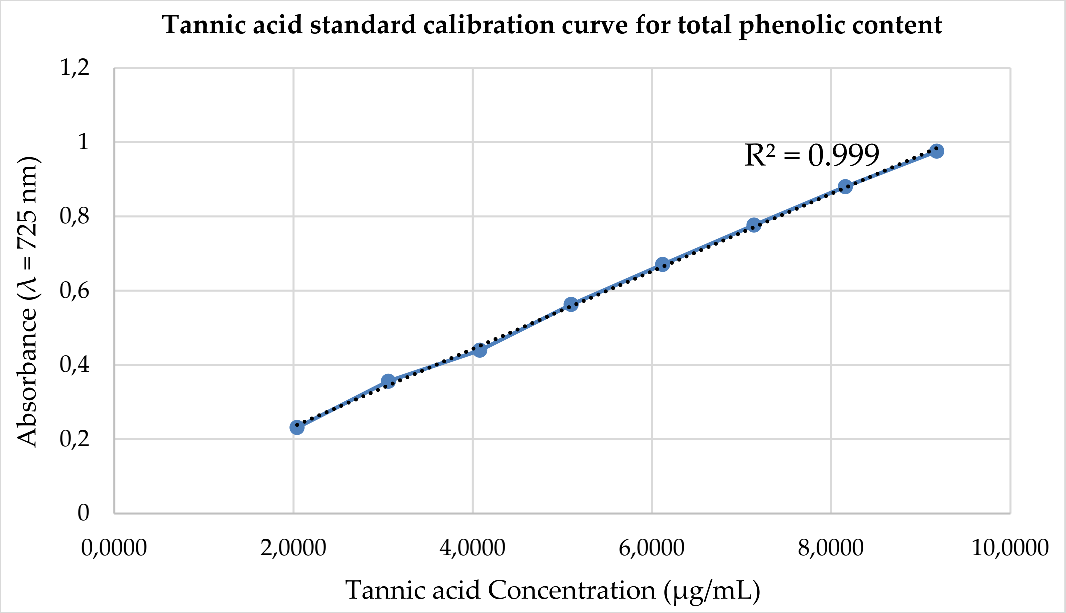

Figure S22. Standard curve for tannic acid (TP).

Optimization of UHPLC and MS conditions

The mobile phase and the flow were investigated for optimal separation of the analytes. According to previous studies, methanol was chosen as the organic part of the mobile phase (La Barbera *et al.*, 2017). A small part of the addition of an organic acid prevents the adaptation to the C18 column of the hydroxyl phenolic moiety (La Barbera *et al.*, 2017).

Polyphenolic compounds have generally shown better mass spectrometric responses to negative ionization (La Barbera *et al.*, 2017; Ciucure and Geană, 2019). Therefore, 0.05% and 0.1% aqueous solutions of formic acid were tested as mobile phase A with a flow rate of 0.3 mL/min. Based on the results, 0.05% formic acid was preferred as mobile phase A. The elution gradient was progressively slowed down in order to increase the number of chromatographic peak data points. The ESI ionization parameters have been optimized to obtain high sensitivity for most compounds. The optimal conditions were presented in the **Materials and methods** section.

Operating in vDIA mode, the instrument alternates between MS and MS2 scans, and a sufficient number of data points per chromatographic peak is required to ensure a large number of detected peaks. In full scan MS, the resolution was set to 70,000 (FWHM at m/z 200), the AGC target was set to 3e6 and the injection time was set to 200 ms. For the MS-MS scan, the resolution was set to 35,000, the AGC target to 1e6 and the injection time to 100 ms. The mass error for all identified compounds was less than 2 ppm.

For the presumptive identification of the compounds without available reference standards, structures were assumed based on the exact mass of deprotonated precursors and ion fragments. The elemental chemical composition for each peak was assigned with a mass error of 2 ppm. The fragmentation pattern was presumed by comparison with NORMAN MassBank (https://massbank.eu/MassBank/), mzCloude Database (https://www.mzcloud.org/), and PubChem (https://pubchem.ncbi.nlm.nih.gov/). Also, ACDLabs MS Fragmenter 2019.2.1 software was used to generate a fragmentation pattern.

Validation of the method for quantifying polyphenols by the UHPLC-HRMS/MS technique

Analytical performances of the target HRMS analysis of polyphenolic compounds from samples were expressed in terms of liniarity, correlation coefficients (R^2), limit of detection (LOD), and limit of quantification (LOQ). The calibration was performed in the concentration range of 0.025 - 2.5 μg/mL for

each of the phenolic compounds by serial dilution with a mixture of water:methanol (90:10) of the stock standard mixture of 1 mg/L. The limit of detection (LOD) and the limit of quantification (LOQ) for each target compound were determined as 3.3 times and 10 times, respectively, the standard deviation of the *y*-intercept, divided by the slope of the calibration curve (ICH Harmonised Tripartite Guideline - Validation of Analytical Procedures: Text and Methodology Q2 (R1)).
The method accuracy was assessed by repeatability and reproducibility evaluation and expressed as relative standard deviations (RSD). The acceptance criterion was an RSD value below 5.0%. Repeatability was assessed by analyzing six samples from the above samples, and reproducibility was examined for three consecutive days with six samples per day. The results were expressed as μg/g of lyophilized plant extract.

Table S8. Validation parameters of the LC-HRMS analytical method.

Compounds/Validation parameters	Calibration equations	R^2	LOD μg/g DW	LOQ μg/g DW	Precision (RSD, %)		Repeatability (RSD, %)
					intra-day	inter-day	
catechin	y=824313.8x+203017.7	0.991	0.40	1.30	3.15	2.85	3.21
epicatechin	y=230287.5x-168641.6	0.992	0.85	2.83	1.41	2.62	2.16
caffeic acid	y=781534.3x+393060	0.990	1.84	6.13	2.36	4.10	3.82
myricetin	y=32881x-738887	0.994	1.21	4.03	0.89	1.36	2.07
p-coumaric acid	y=82377489.4x+486112	0.995	0.62	2.06	1.23	3.04	2.95
syringic acid	y=72565.8x-82305	0.993	0.35	1.16	2.81	4.01	3.87
genistin	y=18732x-110805	0.998	1.65	5.50	1.65	1.85	2.04
chlorogenic acid	y=174329.1x+19222.5	0.998	0.71	2.30	3.45	3.89	4.01
ferulic acid	y=121050x-2321515	0.999	0.15	0.50	1.25	1.87	2.56
hyperoside	y=117573.7x+1512847.8	0.998	0.47	1.56	3.21	4.32	3.58
isorhamnetin	y=3022x+156072	0.999	1.02	3.40	2.47	2.67	2.12
rutin	y=102426.9x+77215.4	0.991	0.65	2.16	2.43	3.16	3.04
gallic acid	y=449675.5x-332050.7	0.994	0.31	1.03	1.26	2.08	2.74
ellagic acid	y=10520x-1437986	0.990	1.03	3.04	1.11	1.15	1.27
formononetin	y=2628478x+16100393	0.999	0.42	1.40	1.48	1.58	2.23
ononin	y=211703.5x+1291087	0.992	10.50	35.10	3.04	4.11	3.98
pinocembrin	y=1301012x-4130476	0.999	0.10	0.30	1.95	2.28	2.65
apigenin	y=897709x-73484551	0.998	0.22	0.73	1.65	2.90	3.07
galangin	y=866716.1x+21534478	0.998	0.15	0.47	1.04	1.87	1.95
pinostrobin	y=695357x-22084171	0.999	0.15	0.50	2.76	3.16	3.82
kaempferol	y=324023.5x+35109689	0.991	0.30	1.00	3.07	3.65	3.18
hesperetin	y=1635.3x-120620	0.990	0.95	3.16	2.89	3.07	3.14
genistein	y=1598102x+5526494	0.999	0.30	1.00	1.58	2.49	2.61
naringenin	y=818977.8x+1767512	0.994	0.14	0.46	2.80	3.74	3.06
naringin	y=73403.1x+53366.8	0.998	6.90	23.00	1.08	2.16	3.85
quercetin	y=125092.1x+747968	0.999	1.60	5.33	1.11	2.65	1.85
glycitein	y=259340.7x+6212357	0.999	0.55	1.83	2.08	3.18	2.74
daidzin	y=1017.2x-74781	0.997	1.08	3.60	2.65	3.74	4.08
daidzein	y=2071x+3009.8	0.999	1.15	3.83	1.43	2.65	2.14
chrysin	y= 430529x-185238	0.995	0.21	0.70	1.08	2.14	2.68
abscisic acid	y=222271x-396923	0.996	0.15	0.37	1.66	1.89	1.54

The correlation coefficient R^2 for all standards was greater than 0.99, showing good linearity. The RSD standard deviation values of the target compounds were less than 5.0%, which proved good accuracy. The values of LOD and LOQ detection and quantification limits were maintained in the range of 0.10 - 10.50 µg/g DW (dry weight) and 0.30 – 35.10 µg/g DW, respectively (**Table S1**).

Table S9. Monitored compounds by full scan - HRMS analysis and MS-MS analysis based on analytical standards.

Compound	Rt (min)	Chemical formula	Exact mass	Error (ppm)	$[M-H]^-$ Ion (m/z)	MS2 Fragment ions (m/z)
			**Flavonoids**			
catechin	7.80	$C_{15}H_{14}O_6$	290.07904	1.47	289.07176	245.08192; 203.07088; 151.03908; 125.02320; 109.02821
epicatechin	10.19	$C_{15}H_{14}O_6$	290.07904	1.25	289.07176	245.08192; 203.07088; 151.03908; 109.02821
quercetin	16.59	$C_{15}H_{10}O_7$	302.04265	0.86	301.03540	245.04601; 178.99809; 273.04059; 121.02814
rutin (quercetin-3-O-rutinoside)	14.20	$C_{27}H_{30}O_{16}$	610.15338	0.50	609.14613	300.02777; 271.02505; 255.02995; 243.02980; 165.01841; 151.00258
apigenin	17.54	$C_{15}H_{10}O_5$	270.05282	1.18	269.04502	227.03389; 181.06430; 151.00194; 149.00226; 117.03271
kaempferol	17.06	$C_{15}H_{10}O_6$	286.04774	0.57	285.04049	255.02977; 201.01866; 151.00262; 107.01250; 92.92660
isorhamnetin	13.20	$C_{16}H_{12}O_7$	316.05830	1.35	315.05105	300.02710; 227.03508; 163.00369; 151.00264; 107.01190
naringenin	19.69	$C_{15}H_{12}O_5$	272.06847	0.50	271.06122	253.05055; 151.00269;

						119.04903; 107.01258
naringin	14.11	$C_{27}H_{32}O_{14}$	580.17920	2.02	579.17185	356.99371; 255.02995; 119.04884
hesperetin	16.83	$C_{16}H_{14}O_6$	302.07904	1.34	301.07179	283.06204; 267.06650; 252.04286; 151.00266; 125.02319
pinostrobin	17.40	$C_{16}H_{14}O_4$	270.08921	1.89	269.08196	254.05864; 210.06839; 177.05495; 148.01559
chrysin	17.63	$C_{15}H_{10}O_4$	254.05791	1.65	253.05066	208.96011; 151.03899; 107.04897; 89.04897; 65.03819
myricetin	10.42	$C_{15}H_{10}O_8$	318.03757	1.37	317.03032	178.99860; 164.92636; 151.00368; 137.02442; 107.01258
galangin	19.98	$C_{15}H_{10}O_5$	270.05282	1.48	269.04557	239.03345; 227.03389; 225.05580; 211.03877; 169.06425
hyperoside (quercetin-3-galactoside)	13.98	$C_{21}H_{20}O_{12}$	464.09548	1.03	463.08768	300.02771; 355.02985; 271.02491; 243.02969; 178.99773; 151.00262
genistin	12.56	$C_{21}H_{20}O_{10}$	432.10565	1.45	431.09837	311.05637; 269.04590; 271.05133; 181.06580
genistein	18.07	$C_{15}H_{10}O_5$	270.05282	1.24	269.04502	159.04420; 133.02835; 201.05527; 181.06546; 107.01257
daidzin	11.42	$C_{21}H_{20}O_9$	416.11073	1.49	415.10348	252.0451; 251.0349; 224.0487; 223.0398
daidzein	16.50	$C_{15}H_{10}O_4$	254.05791	0.87	253.05066	226.05887; 224.04649;

						209.06091; 197.06055; 135.00686; 117.03333
ononin	26.14	$C_{22}H_{22}O_9$	430.12638	2.06	429.11913	355.0969; 341.1109; 267.10280; 252.00778
formononetin	18.74	$C_{16}H_{12}O_4$	268.07356	1.19	267.06631	252.04298; 223.03986; 195.04466; 132.02049
glycitein	16.33	$C_{16}H_{12}O_5$	284.06847	1.18	283.06122	268.0375; 240.0483; 211.03979; 196.05252; 167.02063
			**Phenolic acids**			
gallic acid	1.73	$C_7H_6O_5$	170.02152	0.16	169.01427	125.02318; 141.01823
chlorogenic acid	8.20	$C_{16}H_{18}O_9$	354.09508	0.24	353.08783	192.05876; 191.05544; 173.04474; 127.03876; 85.02806
caffeic acid	8.71	$C_9H_8O_4$	180.04226	0.37	179.03501	135.04390; 107.04881
ferulic acid	14.98	$C_{10}H_{10}O_4$	194.05791	0.62	193.05066	178.02635; 149.05974; 134.03615; 106.04240
ellagic acid	14.44	$C_{14}H_6O_8$	302.00627	1.62	300.99899	185.02349; 283.98961; 229.01391; 157.01006
abscisic acid	15.73	$C_{15}H_{20}O_4$	264.13616	1.42	263.12891	263.12854; 219.13864; 204.11502; 153.09126; 136.05162
p-coumaric acid	10.77	$C_9H_8O_3$	164.04734	0.18	163.03954	211.07640; 135.00754; 119.05020; 17.03320; 116.02670
syringic acid	15.38	$C_9H_{10}O_5$	198.05282	0.41	197.04555	182.02049; 123.00697; 166.99693

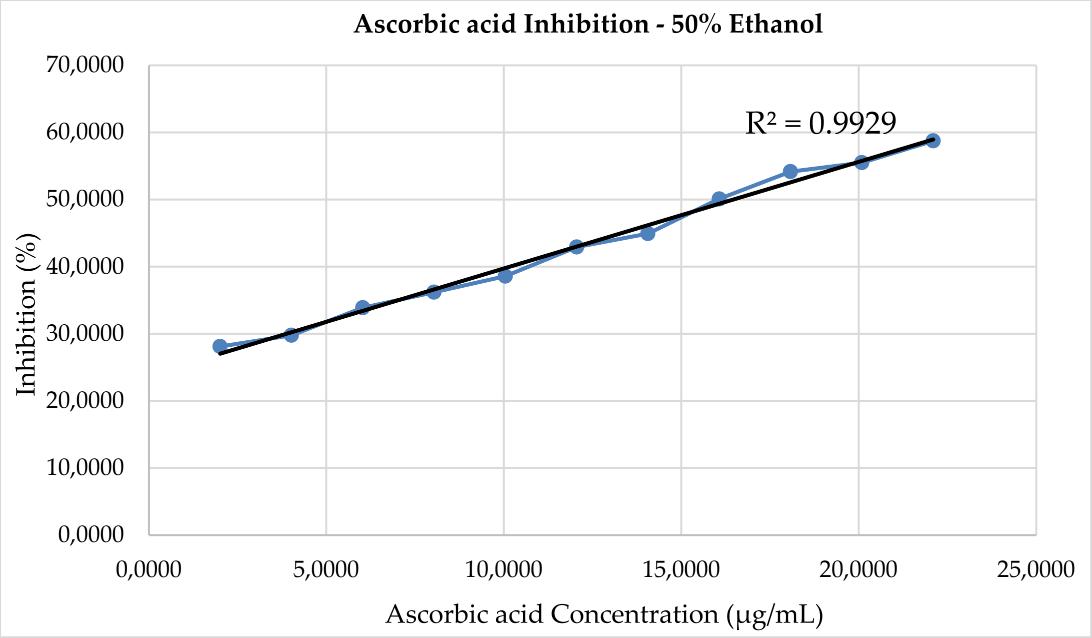

Figure S23. Calibration curve for ascorbic acid (vitamin C) - Antioxidant action in 50% Ethanol.

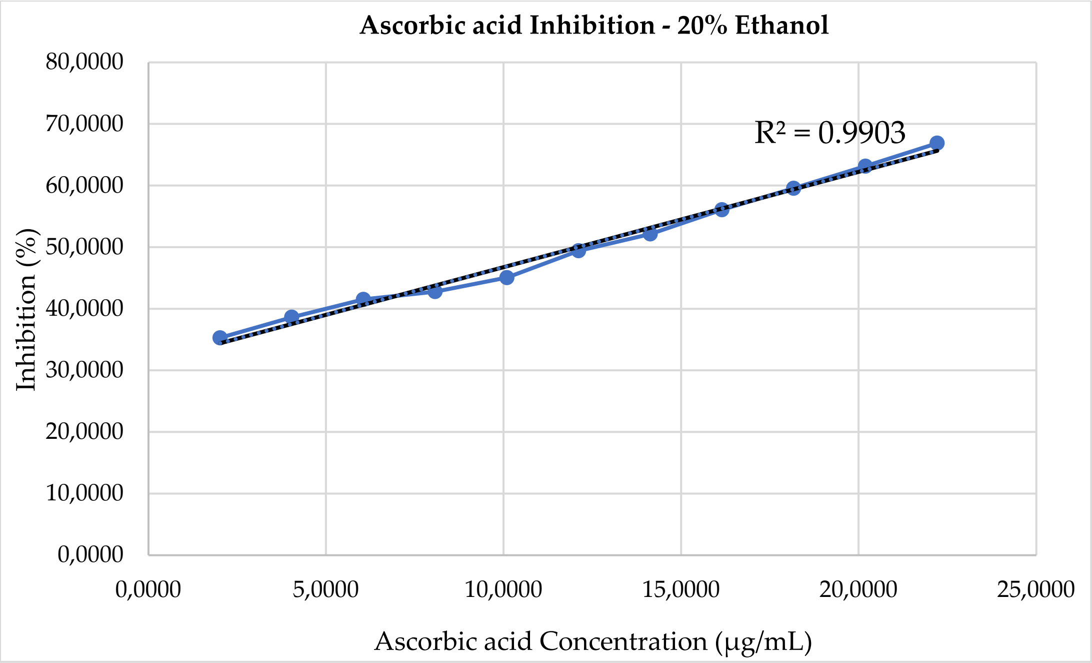

Figure S24. Calibration curve for ascorbic acid (vitamin C) - Antioxidant action in 20% Ethanol.
